# Multivariate PLS Modeling of Apicomplexan FabD-Ligand Interaction Space for Mapping Target-Specific Chemical Space and Pharmacophore Fingerprints

**DOI:** 10.1371/journal.pone.0141674

**Published:** 2015-11-04

**Authors:** Ashalatha Sreshty Mamidi, Prerna Arora, Avadhesha Surolia

**Affiliations:** Molecular Biophysics Unit, Indian Institute of Science, Bangalore, Karnataka, India; University of Copenhagen, DENMARK

## Abstract

Biomolecular recognition underlying drug-target interactions is determined by both binding affinity and specificity. Whilst, quantification of binding efficacy is possible, determining specificity remains a challenge, as it requires affinity data for multiple targets with the same ligand dataset. Thus, understanding the interaction space by mapping the target space to model its complementary chemical space through computational techniques are desirable. In this study, active site architecture of FabD drug target in two apicomplexan parasites viz. *Plasmodium falciparum* (PfFabD) and *Toxoplasma gondii* (TgFabD) is explored, followed by consensus docking calculations and identification of fifteen best hit compounds, most of which are found to be derivatives of natural products. Subsequently, machine learning techniques were applied on molecular descriptors of six FabD homologs and sixty ligands to induce distinct multivariate partial-least square models. The biological space of FabD mapped by the various chemical entities explain their interaction space in general. It also highlights the selective variations in FabD of apicomplexan parasites with that of the host. Furthermore, chemometric models revealed the principal chemical scaffolds in PfFabD and TgFabD as pyrrolidines and imidazoles, respectively, which render target specificity and improve binding affinity in combination with other functional descriptors conducive for the design and optimization of the leads.

## Introduction

Drug Discovery is a complex process, requiring time and money. However, tremendous advances in computational methods have led to versatile approaches like virtual screening, pharmacophore profiling, etc., which hasten the preclinical drug discovery phase. Drug-target recognition is a consequence of binding affinity and specificity, the former governing stability of the complex, while the latter implies discriminating its counter-part from its closely related molecule [1,2]. Conventionally, experimental and computational techniques could determine the binding affinity of a target protein but quantification of binding specificity remains a major challenge. Since, establishing specificity requires relative differences in the binding affinities of the same set of chemical entities with multiple targets, which is often scarce or incomplete; there is a need for computational approaches to compensate for this shortcoming [2,3]. Knowledge from the structural and physiochemical properties of homologous proteins, series of ligands and their interaction advances the traditional drug optimization approaches for an improved drug-target recognition. Thus, virtual screening approach complemented by mathematical modeling using machine learning techniques provide a platform for rapid finding of best hits for prioritizing them as potential leads during the preclinical drug discovery pipeline. In this regard, Lapinsh et al., introduced and improvised proteochemometric analysis (PCM), a machine learning technique involving partial least square modeling for predicting the biological activities and analyzing the receptor-drug interaction space based on physiochemical descriptors of multiple proteins and ligands [4,5]. PCM was successfully employed to study the mode of interaction of G-protein coupled receptors, mutational space of HIV reverse transcriptase and several proteases in the context of drug resistance [6,7,8]. Subsequently, it was implemented to demonstrate its performance and enrichment in virtual screening approaches to find novel small molecule ligands for adenosine receptors [9,10]

*Plasmodium falciparum* that causes malaria in humans and *Toxoplasma gondii*, an opportunistic pathogen causing toxoplasmosis in immune-compromised patients associated with AIDS and congenitally infected infants are the two dreadful parasites of the order apicomplexa. A rapid emergence of resistance in these parasites, unavailability of vaccines against them necessitates a continuous augmentation of the pipeline of molecules to combat these diseases. Both the parasites harbor an endosymbiotic organelle, a vestigial plastid of cyanobacterial origin called apicoplast that possess non-eukaryotic processes and found to be crucial for parasite’s survival. Apicoplast contains all the enzymes of the Type II fatty acid synthetic (FAS) pathway, which are not only essential for the growth of *Toxoplasma* tachyzoites and *Plasmodium* liver stages, but also differ significantly from those of Type I FAS pathway in humans, thus, of interest for drug development against these parasites [11–14]. Some of the earlier studies reported triclosan and thiolactomycin that targeted enzymes of Type II FAS pathway of both these parasites indicating a role of this pathway in their life cycle [15–18]. These studies also identified malonyl CoA: ACP transacylase (FabD) as an important enzyme of Type II fatty acid biosynthetic pathway, which still remains unexplored as drug target in apicomplexan parasites [19–22]. Earlier, we have described pharmacophore profiling to deorphanize FabD in *P*. *falciparum* (PfFabD) [23], and in continuation of that work, we propose a comprehensive approach to quantify the binding affinity and specificity of malonyl CoA: ACP transacylase (FabD) enzyme of apicomplexan parasites through a relative focus on the chemical (drugs) and biologic (target) recognition space with that of host FabDs to aid the development of new therapeutics.

To understand the mechanism of drug-target recognition, the contributions of structural geometries and physiochemical properties to binding affinity were computed. Further, mathematical modeling was performed using partial least square (PLS) method, to ascertain the interaction data consisting of electrostatic (ElecStat) and van der Waal’s (VDW) energy components of their binding free energies to account for their respective interaction space during complexation. These have assisted in understanding the subtle spatial and physiochemical aspects of microscopic environment for high binding affinity and target selectivity of ligands against apicomplexan FabD receptors in the context of other infective and host FabD enzymes.

## Methodology

### Computational infrastructure

Most of the computations were performed in Fujitsu CELSIUS R920 workstation (Fujitsu Technology solutions, Japan). Intensive docking calculations for virtual screening were performed in parallel using the high performance computing Tyrone server (64-core nodes with 2.2 GHz AMD Opteron 6274 processor and 128 GB RAM).

### Construction of 3D models

Homology models of apicomplexan FabD enzymes were built for this study. FabD sequences for *P*. *falciparum* and *T*. *gondii* were retrieved from Uniprot sequence database (www.uniprot.org) using the accession numbers—Q8I6Z9 (403 residues) and V4ZJM0 (502 residues), respectively. Template search in RCSB Protein Data Bank (www.rcsb.org) retrieved FabD of *Escherichia coli* (PDB Id: 2G2Y) and *Vibrio cholera* (PDB Id: 3HJV) with more than 70% coverage and 30% identity against PfFabD and FabD of *E*. *coli* (PDB Id: 2G2Y) and *Staphylococcus aureus* (PDB Id: 3IM9) that exhibited more than 55% coverage and 35% identity against TgFabD. Multiple sequence alignment was performed with query and their respective template sequences using CLUSTALW, set to default parameters. Modeller 9v11 was used to generate homology models based on the sequence alignment and the respective template structures [24]. Three output models were obtained for each PfFabD and TgFabD and the best model was identified using DOPE score. The final models were energy minimized and then subjected to model validation using PROCHECK program of SAVS server (http://services.mbi.ucla.edu/SAVES/).

### Molecular dynamic simulations

Refinement of homology models was performed using molecular dynamic simulations as implemented in GROMACS 4.5.4 [25]. Systems were prepared using CHARMM27 force field and TIP3P water model [26,27]. Initially, the molecular systems were energy minimized in vacuum for 1000 steps employing steepest descent algorithm. Subsequently, periodic boundary conditions were defined by adjusting the boundaries of the cubic box by 10Å. Water and sodium ions were added to the unit cell to maintain overall charge neutrality. Once again, energy minimization was done for 5000 steps to stabilize the solvated systems. Position restrained and unrestrained MD simulations were carried out to equilibrate the solvated system at temperature 300K under 1 bar pressure using Berendesen coupling method [28]. During simulations, LINCS algorithm was applied to constrain all bonds. Electrostatic calculations were accounted by reaction-field with a cut-off distance for Coulomb and van der Waals interactions maintained at 1.4 nm. The final production simulations for each FabD system in free form were run for 30 ns. Subsequently, substrate based optimization of active site environment of FabD was done by carrying out MD simulations in the presence of malonate substrate for 5ns. The topological parameters for malonate were obtained from SwissParam webserver (http://www.swissparam.ch/) [29].

### Virtual Screening

#### Ligand dataset preparation

The ligand dataset used in this study constituted 45,138 compounds obtained from three special subsets of ZINC database (http://zinc.docking.org/browse/subsets/special), namely (i) ZINC drug database (Zdd) comprising commercially drug bank approved drugs and nutraceuticals, (ii) ZINC in man (Zim) containing experimental compounds used for humans and (iii) ZINC natural derivatives (Znd) containing chemically modified natural products. Initially, the compounds were filtered based on nine different parameters viz. molecular weight (32 to 350 g/mol), xlogP (-4 to 3.5), net charge (-5 to 5), number of rotatable bonds (1 to 7), polar surface area (0 to 200 Å^2^), number of hydrogen donors (1 to 10), number of hydrogen acceptors (1 to 20), polar desolvation (-400 to 1 kcal/mol) and apolar desolvation (-100 to 4 kcal/mol). The filtered ligands were checked for redundancy to avoid duplication in the final dataset. Malonyl-thioester-pantothenate was added to the ligand dataset, as it forms the major pharmacophore moeity of FabD substrate and served as a reference compound to select the best hits.

#### Preparation of protein receptors

Six FabD receptors were used in molecular docking calculations with the above filtered ligand dataset. The two apicomplexan FabD models viz. *P*. *falciparum* (PfFabd) and *T*. *gondii* (TgFabD) and four X-ray crystallography structures, which served as templates for homology modeling (i.e. FabD of *V*. *cholerae* (VcFabD), *S*. *aureus* (SaFabD), *E*. *coli* (EcFabD), and *H*. *sapiens* (HsFabD)) were employed. For this, single and complete FabDs were corrected for missing side chains, checked for unnatural amino acids, non-standard atom types and atom occupancy factor using SwissPDB viewer [30].

#### Docking Programs

AUTODOCK 4.2: Initially, docking calculations were done for PfFabD, TgFabD and HsFabD using Autodock 4.2 by implementing a powerful Lamarckian Genetic algorithm for conformational search [31]. FabD receptors were pre-processed by adding Kollman charges, solvation parameters to the atoms and merging the non-polar hydrogens. A 3D grid box was defined based on the four invariant residues in the active site of the respective FabD protein to map the entire binding pocket and generate a grid parameter file by the Autogrid module. Each Autodock cycle or generation consisted of a regimen of fitness evaluation, crossover, mutation, and selection. The GA runs were set to 50 with a step size of 0.2 Å for translations and 5° for torsions, 27,000 generations, 2,50,000 evaluations and clustering analysis with 2.0 Å cut-off. For each run, the estimated free energies of binding, conformations of docked complexes, etc were obtained. The best pose of the ligands that constituted the largest cluster possessing lowest binding energy was selected.

DOCK6: A second level of stringent screening for hit molecules was performed via consensus docking calculations using DOCK 6.6 program (http://dock.compbio.ucsf.edu/DOCK_6/index.htm). The receptors were processed using Dock Prep module of Chimera using AMBER parm99 partial charges and then output in Mol2 format [32]. Active site was identified and prepared by selecting spheres at a distance from 1-10Å from the malonate (substrate molecule). All the input files required to define the negative image of the binding site were prepared to superpose the ligands using the programs present in the DOCK distribution (DMS, SPHGEN, SHOWBOX, and GRID). Ligands were protonated and assigned AM1-BCC charges using the ANTECHAMBER module of AMBER program suite [33–35]. Firstly, rigid docking was done using a geometric matching algorithm followed by refinement through an incremental construction method called anchor-and-grow accounting ligand flexibility using the best orientations from rigid docking. For this, grid-based score was considered, which is based on the intermolecular non-bonded terms viz., van der Waals (VDW) for steric and electrostatic for charge based interactions of the AMBER force field ff99 [36]. The ligand and receptor residues within 8 Å distance from the ligand were rendered flexible to adjust during minimization and MD simulation. Amber MM-GB⁄SA scoring function was then applied on the docked complexes via a thermodynamic cycle to compute the binding free energies, which is calculated as E_Complex_ − (E_Receptor_ + E_Ligand_), and approximated by the Amber force field.

Docking calculations for all the six FabD receptors were considered for generating PLS models.

#### Scoring functions and filters applied

Scoring schemes are crucial to evaluate and re-rank the predicted ligand poses to select the best possible hits during the structure based virtual screening process. The best conformations from AUTODOCK calculations retrieved based on low binding energies were rescored and ranked using X-SCORE to obtain more accurate binding free energies [37]. It implements a consensus scoring function that combines three empirical scoring functions namely HPScore, HMScore, and HSScore to reduce the errors of single score. Following rescoring, binding free energy of malonyl-thioester-pantothenate was used as a threshold to select only those ligands that showed better binding free energies than the FabD susbtrate, but the inverse is true for HsFabD. The next filter applied was based on the pharmacokinetic properties of the ligands to account for their ADME/Tox features, their druggability and toxicity, which was done using FAFDrugs^2^ webserver (http://fafdrugs2.mti.univ-paris-diderot.fr/) [38].

### PLS modeling of FabD interaction space

Four distinct partial least square (PLS) models were generated following the methodology of Lapinsh et al [4]. These models are (i) All-FabDs model that considered all the six FabD receptors, (ii) Pathogen-FabDs model constituting PfFabD, TgFabD, VcFabD and ScFabD, (iii) Apicomplexan-FabDs model comprising PfFabD and TgFabD and the fourth model (iv) Host-FabDs model that contained HsFabD and EcFabD receptors. Furthermore, three chemometric models were developed with PfFabD, TgFabD and HsFabD.

#### Preparation of X-block descriptor dataset for PLS

Calculation of descriptors for FabD receptors: Twenty two non-conserved amino acid residues of the active site of PFabD were mapped using the CASTp calculations (http://sts-fw.bioengr.uic.edu/castp/calculation.php). The corresponding residues lining the binding pocket of the other FabDs were located based on multiple sequence alignment following van Westen et al [10]. Physiochemical descriptors were computed for these amino acids using the five z-scale descriptors (z1-z5) derived by Sandberg et al [39], of which, z1 represents hydrophobicity/hydrophilicity, z2 characterize steric bulk properties and polarizability, z3 signifies polarity and z4 and z5 describes electronic effects of the amino acids. A list of active site residues of all FabDs based on their position corresponding to PfFabD is provided in Table 1.

**Table 1:** List of non-conserved active site residues represented based on the positions of amino acid sequence of *P.falciparum* (PfFabD) and grouped as per the PCM models.

	All FabDs					
Amino acid Positions	Pathogenic FabDs				Host FabDs	
	Apicomplexan FabDs		VcFabD	SaFabD	HsFabD	EcFabD
	PfFabD	TgFabD				
157	SER 157	THR 228	THR 64	THR 67	THR 81	THR 59
192	TYR 192	LEU 266	HIS 96	HIS 96	PHE 116	HIS 91
194	LEU 194	LEU 268	LEU 98	LEU 98	VAL 118	LEU 93
228	LEU 228	SER 302	ALA 132	THR 132	ALA 152	GLU 126
229	TYR 229	ASN 303	GLY 133	GLY 133	VAL 153	GLY 127
231	MET 231	GLY 305	GLY 135	GLY 135	SER 155	GLY 129
232	THR 232	GLY 306	ALA 136	SER 136	GLY 156	ALA 130
233	THR 233	MET 307	MET 137	MET 137	MET 157	MET 131
235	ALA 235	ALA 309	ALA 139	ALA 139	SER 159	ALA 133
262	VAL 262	ALA 345	VAL 164	ALA 165	SER 192	VAL 159
263	SER 263	ASN 346	ASN 165	ASN 166	ASN 193	ASN 160
265	MET 265	LEU 348	ASN 167	ASN 168	LEU 195	ASN 162
271	GLY 271	VAL 354	VAL 173	VAL 174	VAL 201	VAL 168
296	LYS 296	VAL 390	LEU 197	MET 198	ARG 225	LEU 192
297	LYS 297	ARG 391	PRO 198	PRO 199	MET 226	PRO 193
299	GLU 299	LYS 393	PRO 200	ALA 201	PRO 228	PRO 195
300	ILE 300	VAL 394	VAL 201	VAL 202	VAL 229	VAL 196
301	ALA 301	SER 395	SER 202	SER 203	SER 230	SER 197
302	GLY 302	GLY 396	VAL 203	GLY 204	GLY 231	VAL 198
303	ALA 303	ALA 397	PRO 204	PRO 205	ALA 232	PRO 199
304	PHE 304	PHE 398	SER 205	PHE 206	PHE 233	SER 200
359	ILE 359	VAL 453	VAL 260	VAL 261	VAL 288	VAL 255

Description of organic compounds: Signature molecular descriptors of ligands were calculated for PLS modeling. e-DRAGON 1.0 is a webserver (http://www.vcclab.org/lab/edragon/) used for calculating ligand descriptors of different dimensionalities that comprised constitutional descriptors (0D), functional group counts, charge descriptors and molecular properties (1D), topological descriptors (2D) and geometrical descriptors(3D). A total of 68 molecular descriptors were computed for the ligands. For PLS modeling, sixty compounds were considered, whereas for chemometric modeling, the best-hit compounds from virtual screening procedure were also included. The list of descriptors considered for the study is presented in S1 File spreadsheet.

#### Preparation of Y-block dataset for PLS modelling

For PLS modeling, the affinity data in terms of non-bonded interactions (van der Waals and electrostatic energies) and binding free energies (dG) of six FabD receptors and the sixty ligands were included as Y-response variables. Since, interaction energies were considered under Y-block variables, where more negativity indicates higher binding affinity, the absolute values were taken to facilitate linear correlation with the X-block variables.

#### Data slicing

For model creation and prediction, the entire dataset was divided into training and test sets comprising 75% and 25% of the observations, respectively. This is achieved by applying k-means clustering method (where k = 3) on the first four principal components computed using MATLAB (version 7.5, The MathWorks Inc., Natick, MA,) followed by random sub-sampling of the observations to constitute the test set. For generating proteochemometric models, the ligand dataset was divided into 45 and 15 observations comprising working and test sets, respectively, whereas for chemometric models, the fifteen hits shortlisted for PfFabD and TgFabD were included along with the above dataset and divided accordingly.

#### Computing ligand-protein cross terms

Cross-terms were computed for protein and ligand descriptors to account for non-linearity with the binding interactions. Thus, another block of variables comprising cross-terms was constructed containing ligand-ligand (Cl; 68*68), protein-protein (Cp; 110*110) and ligand-protein (Clp; 68*110) cross-terms with 24,204 descriptors.

#### Scaling and variable selection

Before PLS modeling, the dataset was mean centered and scaled to unit variance. As the dataset encompassed different types of descriptors i.e ligand, protein and their cross-terms, hard-block scaling was applied to improve the model. For this, we used scaled weights of N/sqrt, where N is the number of variables in each block. To obtain an optimal model, VIPs (variable importance in projection), which characterize the contribution of X-variables to explain Y responses were subjected to selection by assessing the models iteratively and the insignificant VIPs with values < 1 were excluded.

#### Partial least-squares projections to latent structures (PLS)

In this study, PLS was employed to correlate a matrix of predictor variables, X block (here descriptor data of receptors (Xl block), ligands (Xp block) and cross-terms (Cl, Cp and Clp)) to three response variables constituting Y block. Thus, PLS derives a regression equation which can be expressed as follows:

$$\text{Y }=\bar{\text{Y}}+\;\Sigma \left(\text{coeffl}*\text{xl}\right)\;+\;\Sigma \;\left(\text{coeffp}*\text{xp}\right)\;+\Sigma \;\left(\text{coeffCl }*\text{xl}*\text{xl}\right)\;+\;\Sigma \;\left(\text{coeffCp }*\text{xp}*\text{xp}\right)\;+\;\Sigma \;\left(\text{coeffCl},\text{p }*\text{xl}*\text{xp}\right)$$

The goodness-of-fit of the PLS models was assessed by computing the fraction of explained variation of dependent variables Y (R^2^Y) and predictive Y-variation (Q^2^) through cross-validation, as described previously [40,41,42]. R^2^Y may range between 0 and 1 (where a value closer to unity means better fit) and the value increases upon addition of each extracted PLS component. To rule out the possibility of accumulating chance correlations in the model, Q^2^ values were calculated through seven-fold cross validation and the model was applied on the test set. Concomitantly, a minimum difference between R^2^Y and Q^2^ was maintained and models with R^2^Y > 0.7 and Q^2^ > 0.4 were assumed to be acceptable [43]. Additionally, models were validated by response permutation, wherein, the randomly re-ordered Y-data, R^2^Y and Q^2^ values were re-calculated 100 times and plotted as a function of the correlation coefficient between the original Y and permuted Y. The intercept of the regression line indicates whether the R^2^Y and Q^2^ of original unperturbed model could have been obtained by pure chance [44]. In extension to this, CV-ANOVA was also done to compare two models fitted to the same data by the size of their fitted residuals. F-test is used to test the significance of the null hypothesis of equal residuals of the two models assuming that they are normally distributed. p-value lower than 0.05 asserts the model as significant [45].

All the PLS modeling and analysis was performed using SIMCA 13 software (Umetrics; Singapore). The list of protein and ligand descriptors calculated for all the six FabD models are provided as an excel sheet of supplementary information (S1 File).

## Results and Discussion

**Fig 1:**
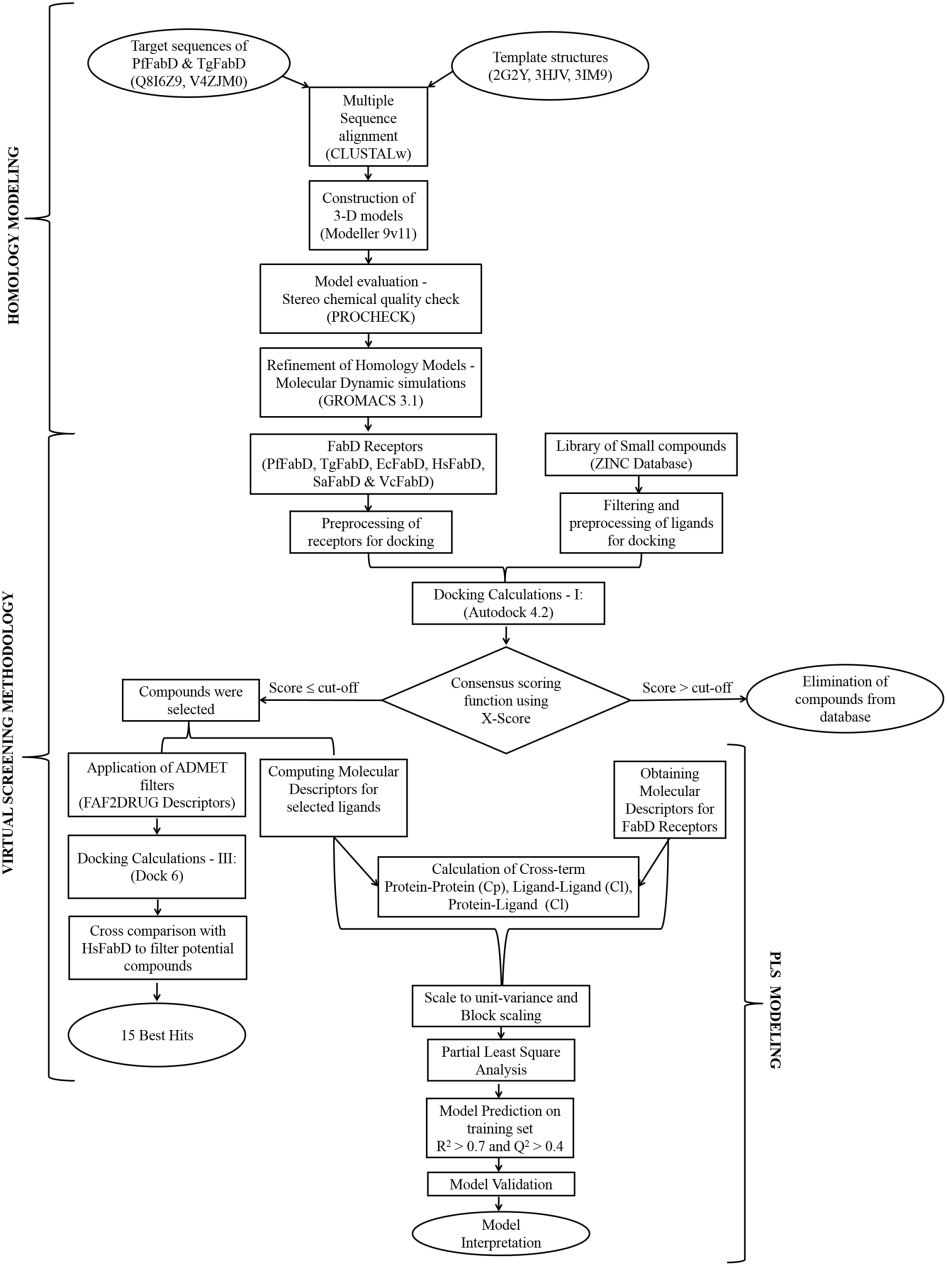
Schematic representation of the workflow carried out for the studies. The methodological process involved homology modeling, virtual screening and PLS modeling to deorphanize FabD drug target of apicomplexan parasites.

While twenty three FabD structures of various bacterial and plant origin have been deposited in PDB, none of them were from any of the apicomplexans. Although, FabD has been proven to be a promising antibacterial target, it still remains unexplored as a drug target for *P*. *falciparum* and *T*. *gondii*. Since, receptor-drug recognition is important for binding specificity and efficacy, micro-level inspection of their interaction space is highly desirable and their determination through experimental methods is time intensive and cumbersome. To aid in such an effort, we have performed virtual screening using the *in silico* models of FabD enzyme of the apicomplexan parasites, generated PLS models using machine-learning techniques to explain the receptor-drug interaction space A flow chart of these studies is presented as Fig 1.

### Exploring FabD of apicomplexan parasites (*P*. *falciparum* and *T*. *gondii*)

Due to the absence of experimentally determined 3D structures of PfFabD and TgFabD, we homology modelled FabDs for this study. Acquiring a significant receptor conformation with accuracy approaching experimentally determined molecular coordinates is critical for the virtual screening process [46]. We have overcome this limitation by constructing substrate bound homology models by supplying distance restraints on the relative orientation of malonate that is already existing in the binding site of EcFabD (PDB ID: 2G2Y) and assigned the coordinates to the target FabD receptors. The backbone coordinates were assigned to PfFabD and TgFabD by transferring the global fold and steric arrangements of the secondary structural elements from template FabDs. The final models selected had relatively low DOPE scores for PfFabD (-38065.1) and TgFabD (-37593.69). During model evaluation, the overall stereochemical quality of the homology models were validated using Ramachandran plots, which are provided as S1 and S2 Figs. For PfFabD, 94.5%, 4.4%, 0.4% and 0.7% residues were found in the most favoured regions, additionally allowed regions, generously allowed regions and disallowed regions, respectively; whereas for TgFabD, the statistics of Ramachandran plot were 90.4%, 5%, 2.5% and 2.1%, respectively. Furthermore, all bond lengths, bond angles and planar groups of main-chain were found to be within limits. Hence, the obtained models are geometrically acceptable.

Homology model mimics the conformations and deformations of the template structures rather than that of the target and hence, optimization of the topology and refinement of internal perturbations in the system was done. Initially, explicit solvent based MD simulations were performed with the homology models in their free forms for stabilizing the molecular conformations. The average RMSDs computed were 0.34±0.04 Å for PfFabD and 0.36±0.04 Å for TgFabD, and is presented as a plot in S3 Fig. Further, MD simulations in the presence of malonate for 5ns were carried out to optimize to comply with global and local stereochemistry of the structures. S4 Fig shows the plot of RMSDs computed as a function of time based on the Cα backbone deviation of both PfFabD and TgFabD and their binding site residues comprising 6Å region around malonate. The average RMSDs noted for PfFabD and TgFabD were 0.18±0.02 and 0.29±0.05 for the entire protein and 0.13±0.02 and 0.12±0.01 for the binding site, respectively. This confirms that the structures of FabD-malonate complexes are stabilized and therefore an optimum conformation can be considered to explore the protein-ligand interaction space.

### Analysis of active site topology of apicomplexans FabDs

**Fig 2:**
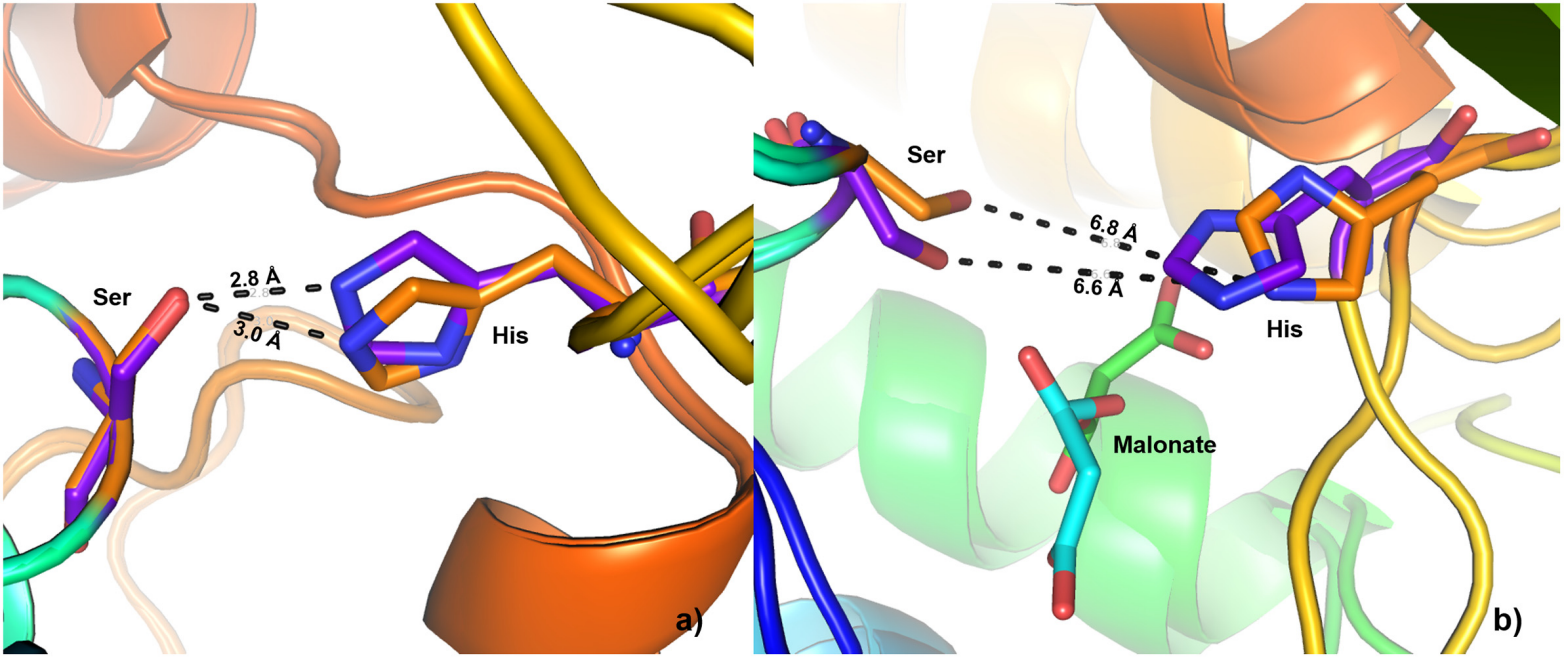
Occurrence of catalytic Ser-His dyad in the binding pockets of PfFabD (represented in purple) and TgFabD (represented in orange). (a) Observed Ser-His dyad in the absence of malonate. (b) disruption of Ser-His dyad in the presence of malonate.

A closer inspection of the two FabDs of apicomplexan parasites revealed a highly conserved architecture consisting of two subdomains; the larger sub-domain exhibited an α/β hydrolase fold and the smaller sub-domain comprizes a four stranded antiparallel β-sheet capped by two short helices, as observed in other bacterial FabDs [47,48]. The active site is a gorge located at the confluence of these two subdomains. The superpositioning of both the apicomplexan FabD structures and their binding pockets are shown in S5 Fig. The two conserved motifs of FabD family: -GQGXQ- and –GXSXG- were noted as –108GQGEQ112- and –191GYSLG195- in PfFabD and -179GQGAQ183- and –265GLSLG269- in TgFabD, respectively. The five key invariant amino acids viz. Q109, S193, R218, H305 and Q354 in PfFabD and Q180, S267, R292, H399 and Q448 in TgFabD corresponding to those reported earlier in the FabDs of *E*. *coli*, *Helicobacter pylori*, *Streptomyces coelicolor*, *Mycobacterium tuberculosis*, etc., were also found to be structurally conserved in PfFabD and TgFabD [47,48,49,50]. The stereochemistry of Ser-His dyad was well preserved through hydrogen bond between the side-chain hydroxyl group of Ser and Nε-2 of His that stabilizes the dyad (Fig 2). The dyad was disrupted upon binding to malonate consistent with the structure of EcFabD, thus endorsing the accuracy of the constructed homology models [49].

**Fig 3:**
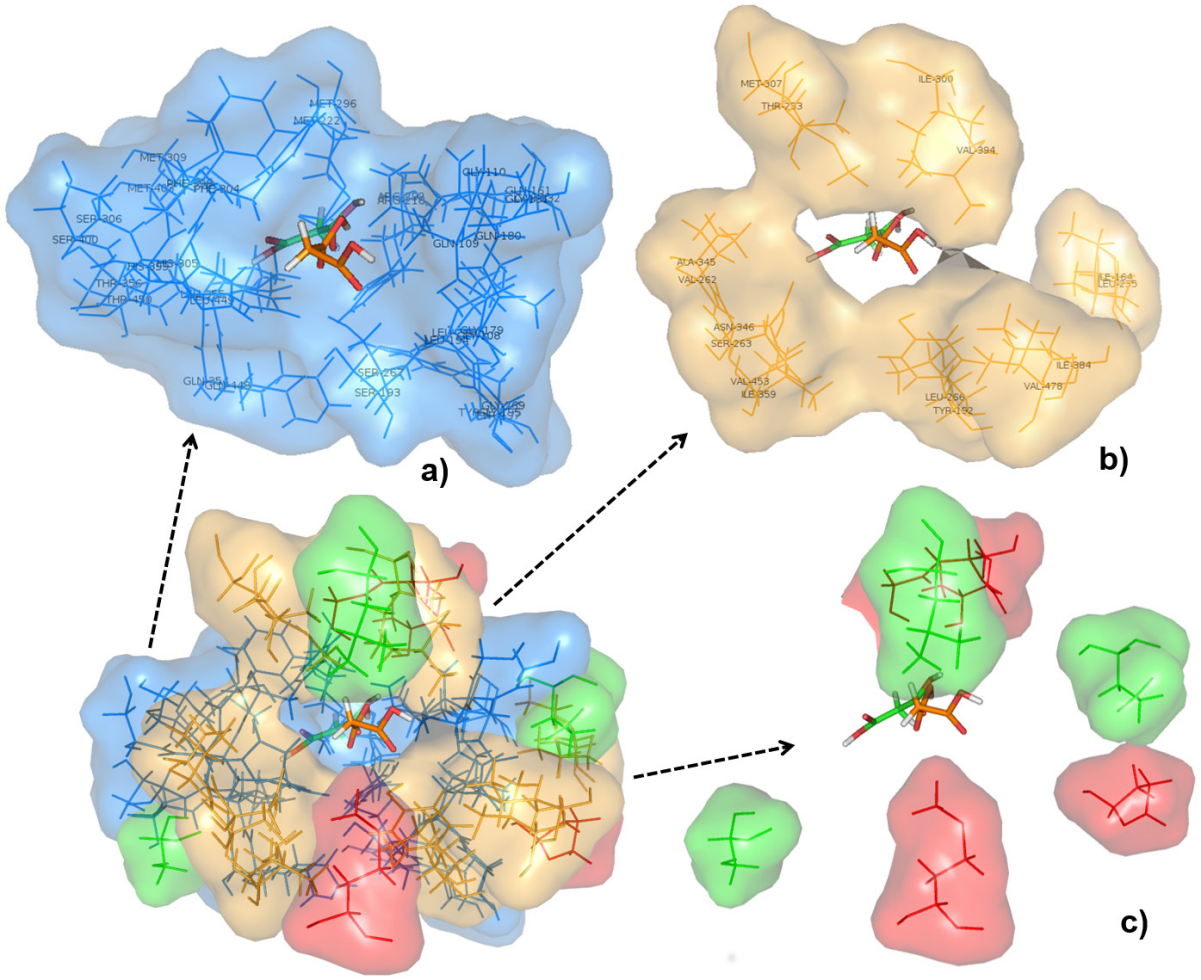
Superposition of PfFabD and TgFabD homology models. Their active site architecture is shown as surface with amino acid residues represented as lines and malonate as sticks. (a) The region of binding site constituting highly conserved amino acid residues are shown in blue color. (b) Residues that exhibited position-specific substitution are shown in orange color. (c) Residues that are unique to PfFabD are shown in red color and TgFabD in green color.

According to Oefner et al., the entire gorge of the EcFabD binding site is involved during malonyl transfer in the presence of holo-ACP [31]. Generally, the binding pocket of FabD harbours two regions—one for specific recognition of malonate and the other for holo-ACP [49,50]. Hence, we investigated in detail the binding pockets of the four FabDs viz. apicomplexan, human and *E*. *coli*, by examining the 8Å region around bound malonate. The binding pockets of apicomplexan FabDs shared 62.07% identities when compared to that of EcFabD or HsFabD. PfFabD exhibited only 27.59% and 51.72% identities and TgFabD showed 56.67% and 66.67% identities with EcFabD and HsFabD, respectively. S6 Fig shows multiple sequence alignment of active site residues of the four FabDs. It was interesting to note that between PfFabD and TgFabD, eighteen amino acid residues located at the base of the gorge connecting the two subdomains were identical, including the five key invariant residues found in FabD family of proteins (Fig 3a). Furthermore, seven position-conserved amino acids substitutions were identified in the holo-ACP binding site surrounding the upper gorge of the active site (Fig 3b), whereas, Glu111, Leu298 and Ser357 in PfFabD and Pro178, Thr228, Ser395 and Met455 in TgFabD located near the entrance of the binding pocket were unique to their respective FabDs (Fig 3c). Similarly, the binding pockets of HsFabD and EcFabD were compared. The latter is a commensal in humans and both together serve as negative models for delineating the relative differences in their active sites from that of the infective agents. While, eighteen amino acids near malonate binding region were identical, ten residues showed position specific substitutions in the ACP binding region adjacent to the entrance of the binding pocket and residues Pro9 and Asn162 in HsFabD and Ser203 and Val229 in EcFabD were unique to them. This indicated that the residues around malonyl-CoA binding site are highly conserved and confer specificity towards selective recognition of the malonate moiety, while the environment of holo-ACP binding site varied in a species-specific manner [51]. S1 Table provides a list of amino acids lining the active sites of these FabDs based on (dis)similarities amongst them.

### Identification of best hits compounds for apicomplexan FabDs

Compounds that are commercially available (Zdd), obtained from natural sources (Znd), or are experimental compounds used in man (Zim) were considered for finding hits against apicomplexan FabDs. For these, three subsets of ZINC database were used, viz. Zdd constituting 2,924 compounds, Znd containing 30,793 compounds and Zim comprising 11,421 compounds were tested for the druggablity of PfFabD and TgFabD. Prior to docking, the large dataset was filtered by defining the acceptable range of physiochemical properties to obtain only the lead-like compounds. Subsequent to the removal of redundant compounds, the final dataset comprised of 14,808 ligands. Since, malonyl-thioester-pantothenate represented the major pharmacophore of FabD susbtrate, it was also included in the final pool of ligands. Consensus scoring functions and filters were applied intermittently with the docking simulations to retain positive candidates exhibiting high affinity as well as good pharmacokinetic properties [52]. Initially, the conformational space available for ligand binding in FabDs was explored using AUTODOCKv4.2. The best pose of each ligand constituting the largest cluster with low binding free energy was retrieved for assessing their binding affinities. Using the emprirical scoring function of X-score, the four energy terms including van der Waals interaction, hydrogen bonding, deformation penalty and hydrophobic effect were predicted for the retrieved FabD-ligand complexes to provide an accurate estimate of binding free energies for rescoring those [53]. The X-score value of malonyl-thioester-pantothenate was used as a threshold for culling only those ligands, which performed better in PfFabD and TgFabD than the substrate, while reverse was true for HsFabD. The cut-off values were determined as 4.51 and 4.73 for PfFabD and TgFabD, respectively and 5.25 for HsFabD. Hence, a total of 3550 and 11683 compounds exhibited better binding with PfFabD and TgFabD than the substrate, respectively. On the other hand, 6195 compounds were retrieved that possessed binding affinities higher than the threshold level i.e. proved inefficient than the malonate-thioester-pantothenate in HsFabD. Consequently, a reverse-match of respective ligand sets of apicomplexan FabDs and HsFabD has led to a total of 160 and 3288 compounds for PfFabD and TgFabD, respectively. Further, a third filter was based on pharmacokinetic properties of the scaffolds. The compounds were selected based on Veber’s, Egan’s and drug-like properties of the orally active drugs, presence of heavy atoms, number of rigid and flexible bonds, TPSA, number and maximum size of system rings and presence of toxic or undesirable substructures. Finally, an ensemble of 60 ligands for PfFabD and 131 ligands for TgFabD passed these ADMET filters.

To achieve an accurate prediction of high quality lead-like scaffolds, multi-stage docking calculations were carried out. Initially shape complementarity method was implemented to explore the geometric shape matching of these filtered ligands inside the binding pocket of FabD receptors, followed by flexible docking via an incremental anchor and grow protocol. The top ranked conformations corresponding to the best-docked energy score for each ligand was selected for rescoring with Amber force field. The average value of Amber scores of each FabD receptor was set as threshold to cross-match the ligands that possessed better score with their respective apicomplexan FabD, but by contrast are insignificant in HsFabD. Thus, fifteen lead-like compounds for each PfFabD and TgFabD were identified that, in addition to their potency, showed target specific binding and possessed drug-like properties. We then checked for significant differences between the binding free energies of shortlisted compounds of the respective apicomplexan FabDs and HsFabD by applying one-way ANOVA F-statistic (α = 5%). The binding affinities varied significantly when compounds specific to PfFabD were also docked to HsFabD (F_(1,28)_ = 10.95; F_crit_ = 4.19) and compounds specific to TgFabD were docked to HsFabD (F_(1,28)_ = 31.02; F_crit_ = 4.19), (see S7a and S7b Fig). The S8a and S8b Fig show the plots of Amber scores of the fifteen shortlisted best hits of PfFabD and TgFabD, relative to HsFabD. The S1 and S2 Schemas provide the details of the fifteen best-hit compounds selected for PfFabD and TgFabD along with their molecular properties, which are well within the range to be categorized as lead-like compounds [54–56]. We note that most of the best-hit compounds were derivatives of natural products, except ZINC00001270, ZINC01529532, ZINC01688939 and ZINC04899687 ligands of PfFabD and ZINC00002159, ZINC03860446 of TgFabD, which are drugs already approved for use in man.

### Exploring protein-ligand molecular interactions

**Fig 4:**
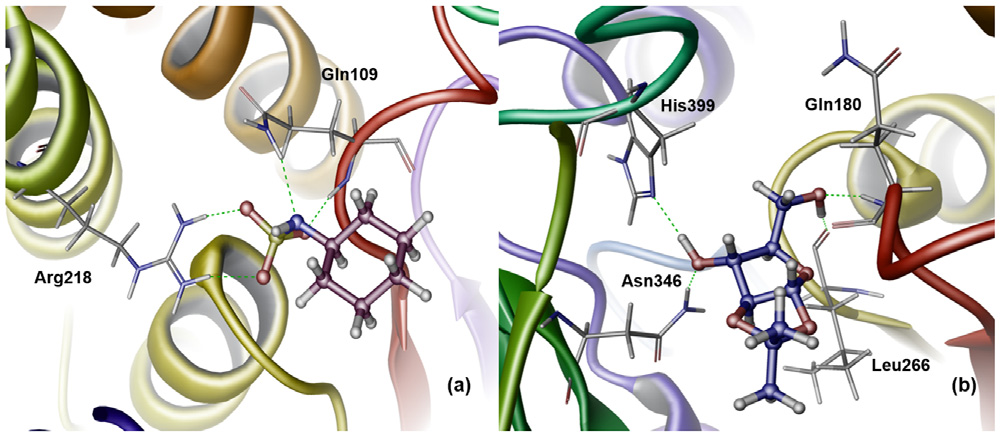
The binding interactions of best hit compounds with apicompolexan FabDs are depicted. Hydrogen bond interactions (green dashed lines) occurring between (a) ZINC01529532 and PfFabD. (b) ZINC00154890 and TgFabD are shown.

Binding affinity and the degree of selectivity towards a target receptor can be attributed to specific intermolecular interactions. Hence, hydrogen bonds, hydrophobic interactions, and π-cation interactions between the ADMET filtered ligands were analysed with their respective apicomplexan FabD targets. The details of various intermolecular interactions in PfFabD and TgFabD are provided in S2 and S3 Tables, respectively. A relative comparison of these molecular interactions with HsFabD that served as negative reference assisted in selecting target specific ligands. S4 and S5 Tables provide the list of residues involved in ligand interactions in HsFabD with the respective hits of PfFabD and TgFabD. In PfFabD, the five compounds viz., ZINC00348080, ZINC00873422, ZINC01529532, ZINC03705320 and ZINC20357942 exhibited relatively better binding affinity and hydrogen bonding interactions with functional residues like Gln109, Tyr192, Arg218 and Ser263, and ZINC03705320 also exhibited π-cation interactions with Phe304. On the contrary, the same ligands proved to have low affinity for HsFabD. Similarly, ZINC00002159, ZINC00154890, ZINC00226411, ZINC02981238, ZINC04343210 and ZINC12955012 showed efficient binding for TgFabD, than to HsFabD. Fig 4 shows the intermolecular interactions of the best hit compounds in the active sites of apicomplexan FabDs.

### PLS modeling for understanding the FabD-ligand interaction space

**Fig 5:**
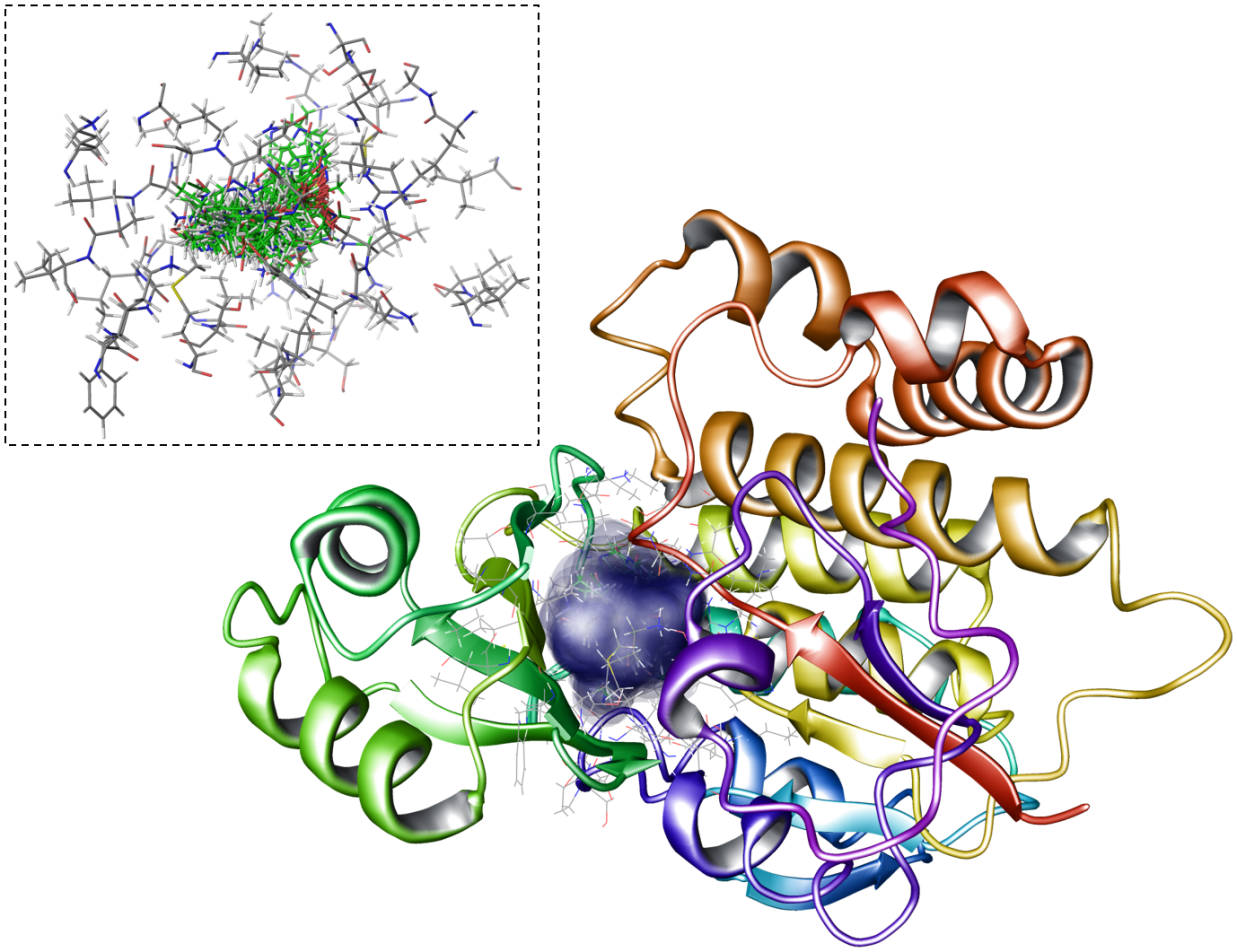
Representaion of the interaction space in PfFabD. The molecular interaction space of FabD protein spanned by sixty ligands obtained from the pipeline of virtual screening process is represented as surface (blue color) and are surrounded by the amino acid residues of active site of PfFabD (shown as inset. The detailed list of active site residues are provided in Table 1.

Till date, PLS modeling has been feasible for only a few drug targets, due to the unavailability of experimentally determined affinity data. However in this study, we made an effort to overcome this drawback by using the interaction data computed through docking simulations in terms of binding free energies and non-covalent interactions like electrostatic (ElecStat) and van der Waal’s (VDW) energies that serve as important recognition forces of molecular complexes [57]. Fig 5 shows the protein-ligand interaction space spanned by the sixty ligands for PfFabD. Four proteochemometric models, viz. All-FabD, Pathogenic-FabD, Apicomplexan FabD and Host-FabD were generated to predict the contributions of different structural and physiochemical properties of FabD receptor space and ligand space. Host-FabDs model comprising HsFabD and EcFabD served as negative model, because both these FabDs co-exist in human body and shared high similarities in their active site architecture. In addition to proteochemometric models, three distinct chemometric models for PfFabD, TgFabD and HsFabD were generated to obtain an inimitable vision of the chemical landscape of the FabD enzyme.

#### Validation of model predictability

**Fig 6:**
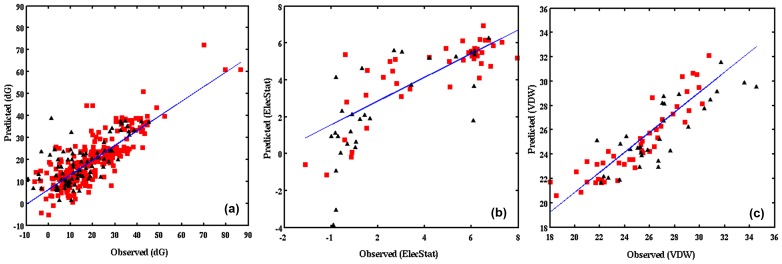
The correlation plots of observed and predicted values derived from the PLS models. (a) All-FabD, (b) TgFabD, (c) PfFabD based on dG, ElecStat, VDW Y-response variables, respectively are shown. The goodness of fit between the observed and predicted values are indicated by the regression lines for training (red) and test (black) sets.

The prospective capabilities of these PLS models were assessed by a combination of cross validation and response permutations [58]. Table 2 provides the complete details of the statistical metrics for validation of the seven models. The robustness of the induced models was explored from the seven-fold cross validation of the training set and their respective R^2^Y and Q^2^ values, which were found to be above 0.7 and 0.4 [43]. Also, over-fitting of the models were tested by calculating the response permutations for R^2^Y and Q^2^ values through their intercepts iR^2^ and iQ^2^, which were smaller than R^2^ value and below zero, respectively [44]. Thus, ruling-out the possibility that the models were predicted merely by chance [44]. Also, CV-ANOVA for individual Y-response variables were performed and found that the p-values were lesser than 0.05 for most of the models. However, significant correlation with VDW was not observed in Pathogen-FabD, Apicomplexan-FabD and TgFabD models [45]. This indicated the inability of X-descriptor variables to accurately predict the variations in VDW interactions. Additionally, to confirm the accuracy and reliability of the PLS models, we have evaluated the external predictability of these models using test sets that constituted one-fourth of the total compounds that were excluded from the training set. The models were regarded as significant, if the Q^2^ext was ≥ 0.4 for at least two of the response-variables. The correlation of predicted versus observed values of dG in All-FabD, VDW in PfFabD and ElecStat in TgFabD models are shown in Fig 6. In chemometric models the variations in dG and VDW Y-response variables were not well-explained by the variance in X-descriptor variables, whereas prediction using Y-ElecStat were found to be more accurate and reliable. Considering the individual R^2^ and cumulative R^2^Y and the overall predictability (Q^2^ values ≥ 0.4) of training set, we conclude that it is appropriate to interpret the PCM models based on dG and ElecStat, while only the latter was satisfactorily analyzed for chemometric PLS models.

**Table 2:** Details of the seven PLS models generated for FabD receptors and statistical metrics for model validate.

Models	Components	R^2^X	R^2^Y	Q^2^	Y-response variables	Response permutations		p-value CV-ANOVA	R^2^ Training	Q^2^ext
						R^2^	Q^2^			
					dG	0.13	-0.43	4.42E-27	0.67	0.41
All-FabDs	10	0.87	0.7	0.42	ElecStat	0.12	-0.47	9.49E-38	0.69	0.55
					VDW	0.14	-0.4	0.003475	0.73	0.33
					dG	0.33	-0.47	8.81E-20	0.75	0.52
Pathogen-FabDs	7	0.5	0.75	0.43	ElecStat	0.34	-0.47	1.79E-18	0.76	0.64
					VDW	0.33	-0.4	0.0488799	0.73	0.17
					dG	…	…	…		
Host-FabDs	6	0.78	0.7	0.42	ElecStat	0.25	-0.59	0.004158	0.69	0.49
					VDW	0.23	-0.63	3.45E-08	0.94	0.95
					dG	0.41	-0.62	1.35E-07	0.84	0.54
Apicomplexan-FabDs	7	0.64	0.82	0.42	ElecStat	0.4	-0.61	4.55E-10	0.83	0.65
					VDW	0.31	-0.39	0.0474655	0.8	0.09
					dG	0.66	-0.48	0.08411	0.62	0.35
PfFabD	6	0.49	0.89	0.57	ElecStat	0.65	-0.57	0.000867	0.77	0.55
					VDW	0.65	-0.59	0.000762	0.82	0.73
					dG	0.34	-0.14	0.001702	0.75	0.22
TgFabD	3	0.48	0.76	0.41	ElecStat	0.3	-0.07	0.0164834	0.64	0.62
					VDW	0.33	-0.11	0.18268	0.91	0.01
					dG	…	…	…		
HsFabD	5	0.4	0.92	0.54	ElecStat	0.69	-0.46	0.126547	0.79	0.61
					VDW	0.69	-0.54	3.34E-05	0.65	0.28

#### Model based interpretation of target and ligand space

To interpret the biologic and chemical space in terms of molecular and physiochemical descriptors of FabD active site and small organic compounds, we performed an in depth analysis of the PLS models. S6 and S7 Tables list the various ligand and protein descriptors that are capable of explaining the variance in the seven PLS models.

**Fig 7:**
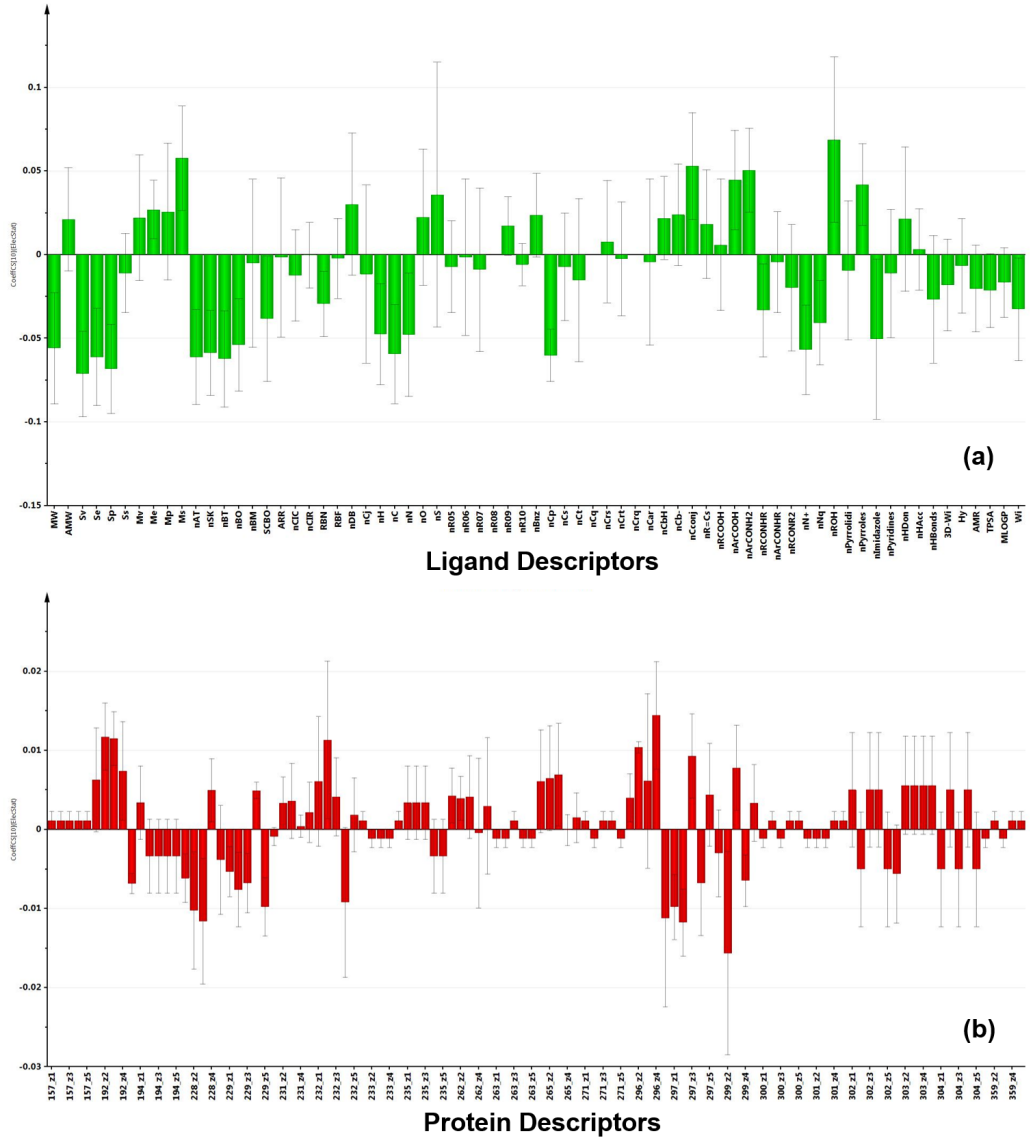
This figure show the coefficient plots depicting the relation between the ElecStat Y-response variable and the (a) ligand descriptors (b) physiochemical properties of amino acid residues in the active site of PfFabD enzyme.

All-FabD PLS model: Taken together the six target proteins provided a comprehensive picture of the interaction space in FabDs. This model explains the variation in dG (Q^2^ext = 0.41) and ElecStat (Q^2^ext = 0.55) parameters. Fig 7a and 7b representing the PLS regression coefficient plots shows the influence of different ligand and protein descriptors on the variation in ElecStat Y-response variable, respectively. Based on the ligand descriptors, organic compounds with nR09, nPyrroles, and nROH as functional groups and sulphur atoms (nS) are important for electrostatic interactions (ElecStat) as well as for the affinity (dG). Contribution of other descriptors is depicted as column plots in S9 Fig. Similarly, the active site residues at positions 157, 229, 231, 232, 233, 263, 265, 271, 297, 300, 301 and 359 (see Table 1 of methodology section) contribute to the binding efficacy and electrostatic interactions in FabDs. Interestingly, these amino acids are common in at least three of the six FabD receptors, which confirms that this model presents the global features of the FabD-ligand interaction space.

Apicomplexan FabD PLS model: Since, the apicoplast in *P*. *falciparum* and *T*. *gondii* is of bacterial origin and all of them possess Type-II fatty acid biosynthetic pathway [59], the apicomplexan, and pathogenic bacterial FabDs were integrated together in a group for modelling (Pathogen-FabDs model). The PLS regression model was satisfactory in terms of dG (Q^2^ext = 0.52) and ElecStat (Q^2^ext = 0.64), S10 Fig provides the contributions of ligand descriptors that positively influenced the binding affinity. The ligands constituting ring structures (nCIC), especially pyridines, pyrroles, nR06, nR10, nCconj, nS, RBF, nROH were noticed to enhance the binding affinity and electrostatic interactions. Since, the contributing amino acid residues were found to be same as for All-FabD model, we analyzed the interaction space of apicomplexan specific FabD. The variance in dG and ElecStat response variables was well explained by the X-descriptor variables with Q^2^ext of 0.54 and 0.65, respectively. nPyrroles, nPyridines, nR06 and nR09 among ring descriptors, nS, nO, nDB, Ms of constitutional indices, nROH, nCrs, nCrt, nRCOOH, nHAcc, nCconj, nArCONHR and nCb- as functional groups and others being Molar Refractivity (AMR), Moriguchi Octanol-water Partition Coefficient and Wiener Index (WI) were noted as important to ligand descriptors specific to apicomplexan FabDs. S11 Fig shows the descriptors of ligands that had an impact on the binding efficacy in PLS model. When the receptor space was analyzed, five amino acids at positions 194, 235, 302, 303 and 304 were found to be identical in apicomplexan FabDs, while amino acids at positions 194 and 235 were common in all Pathogen-FabD model. Further, in addition to the twelve active site residues of All-FabD model, amino acids at positions 228 and 297 also contributed to the interaction space of Apicomplexan FabDs.

Host-FabDs PLS model: Here, FabDs of human and *E*. *coli* were included under one group in view of their binding site similarities as discussed before and because *E*. *coli* inhabits human body as a commensal. Unlike other PLS models, Host-FabDs model was better predicted using only two Y-response variables, i.e. ElecStat and VDW and inclusion of dG did not generate reliable model. Hence, model was generated using six PLS components with R^2^Y = 0.7, Q^2^ = 0.42 and Q^2^ext = 0.49 and 0.95 for ElecStat and VDW Y-responses, respectively. Further, model interpretation revealed the contributing structural and functional descriptors of ligands, which participate in non-covalent interactions for this model. These included nPyridines, nPyrroles, nBnz, nR06, nCIR as ring descriptors; nArCONH2, nArCONHR, nCrs as functional groups; Ms, Mv, nS, nC, RBF as descriptors of constitutional indices, and Molar Refractivity (AMR). S12 Fig presents the coefficient plot of ElecStat and VDW for these ligand descriptors that enchance the binding affinity for the Host-FabD model. Subsequently, upon examining the receptor space, we found that aminoacid residues—Thr, Met, Asn, Val, Pro, Val, Ser and Val at positions 157, 233, 263, 271, 299, 300, 301 and 359, respectively, were the same in both HsFabD and EcFabD. Hence, variance in ElecStat and VDW response variables were correlated using residues at positions 192, 194, 228, 229, 231, 232, 235, 265, 296, 302, 303 and 304.

A comparative analysis of the chemical space of Apicomplexan-FabD and Host-FabD models revealed common descriptors in both the models, i.e. nPyrroles, nR06, nS, nCrs, nArCONHR, AMR and Ms. While, the former differed from the latter by descriptors like nPyrrolidines and nR09 of ring systems, nROH, nRCOOH, nHAcc, nCconj, nCrt of functional group counts, nDB and nO belonging to constitutional indices and others like MLOGP and WI. The uniqueness of Host-FabD model therefore can be attributed to the presence of nPyridines, nBnz, nARCONH2, Mv, nC, RBF and nCIR as ligand descriptors.

#### Computing chemometric PLS models for individual FabDs

**Fig 8:**
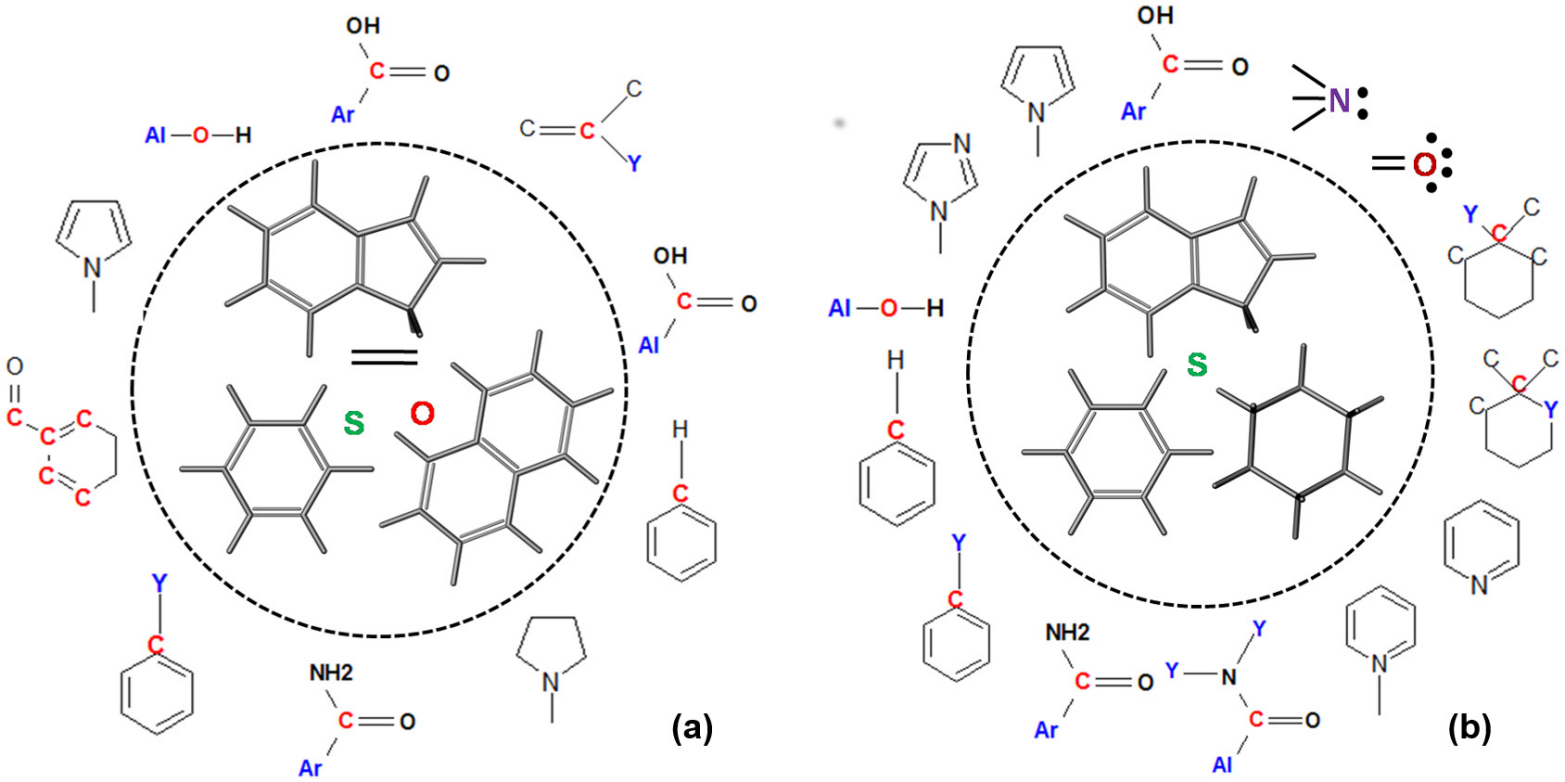
The key structural and functional descriptors obtained through PLS modeling of chemical space in (a) PfFabD and (b) TgFabD. These are crucial for rendering target-specificity of organic compounds are shown. The constitutional indices that form the major scaffolds are enclosed in a circle (dashed line----) and the functional groups are presented around it.

Understanding the structural and other physiochemical features of ligands is critical for designing drug molecules with proper functional groups. Hence, chemometric PLS models of the chemical space in PfFabD and TgFabD with respect to HsFabD were developed. In the chemometric PLS models, the cumulative R^2^Y and Q^2^ were above the threshold range, i.e. 0.7 and 0.4, while the Q^2^ext for ElecStat has alone qualified with ≥0.4. On the other hand, Q^2^ext values of dG and VDW were lesser than 0.4. The entire descriptor details corresponding to the three Y-dependent variables has been provided in S6 Table, in view of the above, these models are discussed in the context of ElecStat Y-response variable only. Based on these models, the ligand space that was conducive for the specificity for PfFabD and TgFabD are shown as column plots in S13 Fig. Further, analysis of the descriptors, which describe the organic compounds and their functional groups that enhance the binding interactions with respective apicomplexan FabD receptors are shown in Fig 8. Upon comparison with HsFabD, it is noticed that average molecular weight (AMW), mean atomic Sanderson electronegativity (Me), nCrs, nPyrrolidines, nArCONH2 as functional groups, MLOGP and Weiner index (WI) render specificity of the given ligands for PfFabD (S12a Fig). Similarly for TgFabD, ligand features influencing selectivity are AMW, Me, RBF, SCBO, nCIC, ARR, nBM, nCar, nRCONR2, nArCONH2, nImidazole, MLOGP (S12b Fig). Likewise, the contributing chemical descriptors that positively influence the electrostatic interactions of HsFabD are shown as column plot in S14 Fig. The chemical space specific to HsFabD relative to PfFabD was assessed and noted to consist of nCt, nCrt, nPyridines, hydrophilicity and number of hydrogen donor atoms (N and O). Similarly, functional groups like nRCOOH, nARCONHR, nCconj, nCrt, nDB, nO, nR = Cs, nR10, nPyrrolidines and hydrophilicity factor (Hy) specifically contribute for HsFabD in competition with TgFabD.

**Fig 9:**
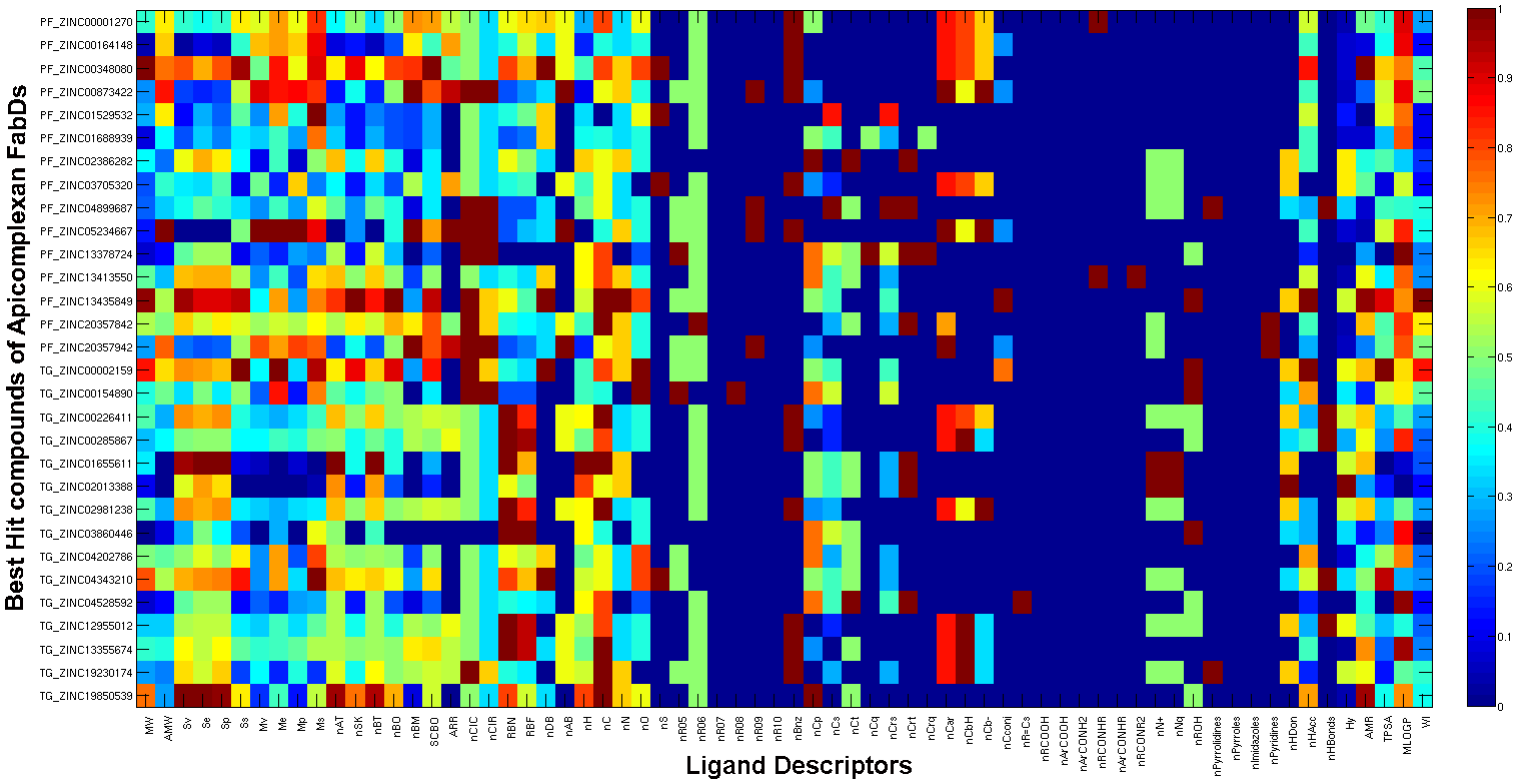
Heatmap of the ligand descriptors representing the diverse chemistry of the best hit compounds obtained for PfFabD (compounds with suffix ‘PF’) and TgFabD (compounds with suffix ‘TG’) via the virtual screening process.

Thus, the above description of the chemical space that positively enhance target selectivity and at the same time show less or no impact on HsFabD were taken into consideration for lead optimization and improvement of binding specificity and efficacy. We then extrapolated the above models to find the contributing factors of the best-hit compounds obtained through virtual screening process for PfFabD and TgFabD. The diverse chemistry of these molecules depicted as a heatmap for all the thirty leads is presented in Fig 9. Most of these compounds lacked the ring structures like pyrroles, pyrrolidines, imidazoles, etc., and sulphur atoms, which add to their target specificity.

## Conclusions

In this work, we advance an idea for the preclinical drug discovery process to identify target specific inhibitors for FabD enzymes of apicomplexan parasites through virtual screening and mapping the pharmacophore space. PLS modeling of receptor-ligand interaction space in PfFabD and TgFabD in comparison with HsFabD has highlighted the amino acid environment crucial for binding site and the complementary chemical space in terms of constitutional, topological, functional and other molecular property descriptors. This combinatorial approach demonstrates the added value of mathematical modeling based on machine learning approaches to illustrate its efficiency for finding ligands that are target specific and identifying the specific pharmacophore fingerprints capable of improving binding affinity. We hope that experimental validation of this approach will expand its scope for application to other unexplored drug-targets.

## Supporting Information

S1 Schema Fifteen shortlisted ligands as lead compounds for PfFabD. (DOCX)

S2 Schema Fifteen shortlisted ligands as lead compounds for TgFabD. (DOCX)

S1 Table Presenting the active site residues of Apicomplexan and Host FabDs for a relative comparison. (DOCX)

S2 Table Details of the amino acids participating in various intermolecular interactions with the shortlisted hits specific to PfFabD. (DOCX)

S3 Table Details of the amino acids participating in various intermolecular interactions with the shortlisted hits specific to TgFabD. (DOCX)

S4 Table Inter-molecular interactions of HsFabD with the shortlisted leads of PfFabD. (DOCX)

S5 Table Inter-molecular interactions of HsFabD with the shortlisted leads of TgFabD. (DOCX)

S6 Table List of structural and physiochemical ligand descriptors contributing positively towards binding affinity of different PLS models. (DOCX)

S7 Table List of protein z-scale descriptors contributing positively towards binding affinity of different PLS models. (DOCX)

S1 Fig Ramachandran plot of PfFabD homology model used for model evaluation. (TIF)

S2 Fig Ramachandran plot of TgFabD homology model used for determining model accuracy. (TIF)

S3 Fig The RMSD computed for 30ns MD trajectory obtained during model refinement for PfFabD (black) and TgFabD (red). (TIF)

S4 Fig RMSD plots calculated as a function of time for both PfFabD and TgFabD. (i) the Cα-backbone coordinates represented in black and red colors, respectively, and (ii) binding residues spanning 6Å region around malonate in its binding pocket represented in green and blue colors, respectively.
(TIF)

S5 Fig Superposition of PfFabD (purple) and TgFabD (orange) models (represented as cartoons) with malonate (shown as sticks) in their binding pockets. (TIF)

S6 Fig Sequence alignment of binding site amino acid residues surrounding the 6 Å region lining the active site of the four FabDs viz *P*. *falciparum* (PF); *T*. *gondii* (TG); *E*. *coli* (EC) and *H*. *sapiens* (HS). (TIF)

S7 Fig A notched boxplot depicting the binding free energies computed for the best-hit compounds of each Apicomplexan FabDs incomparison with HsFabD; (a) PfFabD and (b) TgFabD. (TIF)

S8 Fig Bar plots representing the Amber scores calculated for the fifteen best hits of PfFabD (a) and TgFabD (b) relative to HsFabD. (TIF)

S9 Fig Coefficient Plots of X-descriptors correlated with dG and ElecStat of All-FabD PLS model. (TIF)

S10 Fig Coefficient Plots of X-descriptors correlated with dG and ElecStat of Pathogen-FabD PLS model. (TIF)

S11 Fig Coefficient Plots of X-descriptors correlated with dG and ElecStat of Apicomplexan-FabD PLS model. (TIF)

S12 Fig Coefficient Plots of X-descriptors correlated with dG and ElecStat of Host-FabD PLS model. (TIF)

S13 Fig The column plots showing the regression coefficients of various ligand descriptors that contribute positively towards electrostatic interaction energies for an enhanced binding affinity of (a) PfFabD and (b) TgFabD. (TIF)

S14 Fig The column plot showing the regression coefficients of various ligand descriptors that contribute positively towards electrostatic interaction for an enhanced binding affinity of HsFabD. (TIF)

S1 File Spreadsheet containing the data used in this study. (XLSX)
