# Supplementary material for: Multivariate PLS Modeling of Apicomplexan FabD-Ligand Interaction Space for Mapping Target-Specific Chemical Space and Pharmacophore Fingerprints
Source: PLoS One. 2015 Nov 4;10(11):e0141674. doi: 10.1371/journal.pone.0141674 (PMC4633102; doi:10.1371/journal.pone.0141674)
Supplement: S1 Schema — (DOCX) [file pone.0141674.s001.docx]

| 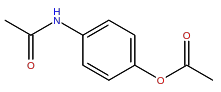 | | 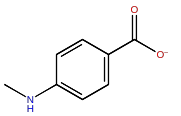 | | 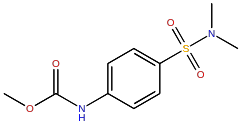 | |
| --- | --- | --- | --- | --- | --- |
| **(i) ZINC00001270** | | **(ii) ZINC00164148** | | **(iii) ZINC00348080** | |
| 4-(acetylamino)phenyl acetate | | 4-(Methylamino)benzoic acid | | methyl [4-(dimethylsulfamoyl) phenyl]aminoformate | |
| MW = 193.20 | RB = 3 | MW =150.15 | RB = 2 | MW = 258.29 | RB = 4 |
| tPSA = 55 | HA = 4 | tPSA =52 | HA =3 | tPSA = 76 | HA = 6 |
| xlogP = 0.71 | HD = 1 | xlogP =1.71 | HD =1 | xlogP = 0.96 | HD = 1 |
| 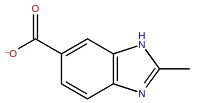 | | 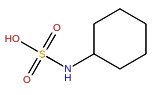 | | 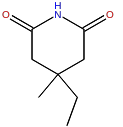 | |
| **(iv) ZINC00873422** | | **(v) ZINC01529532** | | **(vi) ZINC01688939** | |
| 2-methyl-1H-benzimidazole-5-carboxylic acid | | Cyclamic acid | | 3-Ethyl-3-MethylglutariMide | |
| MW = 175.16 | RB = 1 | MW = 178.23 | RB = 2 | MW = 155.19 | RB = 1 |
| tPSA = 69 | HA = 4 | tPSA = 69 | HA = 4 | tPSA = 46 | HA = 3 |
| xlogP = 1.40 | HD = 1 | xlogP = -0.94 | HD = 1 | xlogP = 0.64 | HD = 1 |
| 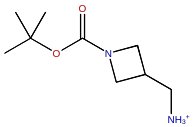 | | 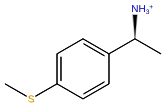 | | 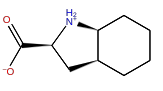 | |
| **(vii) ZINC02386282** | | **(viii) ZINC03705320** | | **(ix) ZINC04899687** | |
| tert-Butyl 3-(aminomethyl)azetidine-1-carboxylate | | 1-[4-(Methylthio)phenyl]ethanamine | | (2S,3AS,7AS)-2-Carboxyoctahydroindole | |
| MW = 187.26 | RB = 3 | MW = 168.28 | RB = 2 | MW = 169.22 | RB = 1 |
| tPSA = 57 | HA = 4 | tPSA = 27 | HA = 1 | tPSA = 57 | HA = 3 |
| xlogP = 0.41 | HD = 3 | xlogP = 0.24 | HD = 3 | xlogP = 0.68 | HD = 2 |
| 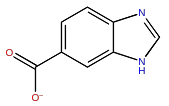 | | 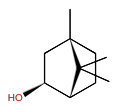 | | 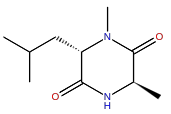 | |
| **(x) ZINC05234667** | | **(xi) ZINC13378724** | | **(xii) ZINC13413550** | |
| 1H-Benzimidazole-5-carboxylic acid | | (1S,2S,4S)-4,7,7-trimethylnorbornan-2-ol | | (3R,6S)-6-isobutyl-1,3-dimethyl-piperazine-2,5-dione | |
| MW = 161.14 | RB = 1 | MW = 154.25 | RB = 0 | MW = 198.26 | RB = 2 |
| tPSA = 69 | HA = 4 | tPSA = 20 | HA = 1 | tPSA = 49 | HA = 4 |
| xlogP = 1.32 | HD = 1 | xlogP = 2.35 | HD = 1 | xlogP = 0.60 | HD = 1 |
| 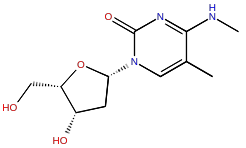 | | 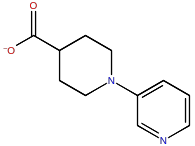 | | 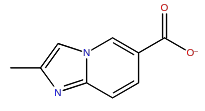 | |
| **(xiii) ZINC13435849** | | **(xiv) ZINC20357842** | | **(xv) ZINC20357942** | |
| 1-[(2S,4S,5S)-4-hydroxy-5-(hydroxymethyl)tetrahydrofuran-2-yl]-5-methyl-4-methylamino-pyrimidin-2-on | | 1-(Pyridin-3-yl)piperidine-4-carboxylic acid | | 2-Methylimidazo[1,2-a]pyridine-6-carboxylic acid | |
| MW = 255.27 | RB = 3 | MW = 205.23 | RB = 2 | MW = 175.16 | RB = 1 |
| tPSA = 97 | HA = 7 | tPSA = 56 | HA = 4 | tPSA = 57 | HA = 4 |
| xlogP = -0.90 | HD = 3 | xlogP = 0.99 | HD = 0 | xlogP = 1.05 | HD = 0 |

**S1 Schema. Fifteen shortlisted ligands as lead compounds for PfFabD**
