# Supplementary material for: Multivariate PLS Modeling of Apicomplexan FabD-Ligand Interaction Space for Mapping Target-Specific Chemical Space and Pharmacophore Fingerprints
Source: PLoS One. 2015 Nov 4;10(11):e0141674. doi: 10.1371/journal.pone.0141674 (PMC4633102; doi:10.1371/journal.pone.0141674)
Supplement: S2 Schema — (DOCX) [file pone.0141674.s002.docx]

| 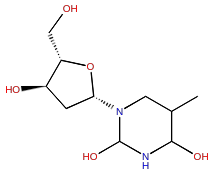 | | 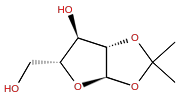 | | 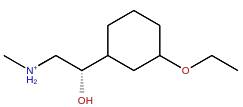 | |
| --- | --- | --- | --- | --- | --- |
| **(i) ZINC00002159** | | **(ii) ZINC00154890** | | **(iii) ZINC00226411** | |
| 1-(2-Deoxy-beta-L-erythro-pentofuranosyl)-5-methylpyrimidine-2,4(1H,3H)-dione | | 1,2-O-Isopropylidene-D-xylofuranose | | 1-(3-Ethoxy-phenyl)-2-methylamino-ethanol | |
| MW = 242.23 | RB = 2 | MW = 190.19 | RB = 1 | MW = 196.27 | RB = 5 |
| tPSA = 105 | HA = 7 | tPSA = 68 | HA = 5 | tPSA = 46 | HA = 3 |
| xlogP = -1.43 | HD = 3 | xlogP = 0.41 | HD = 2 | xlogP = 1.32 | HD = 3 |
| 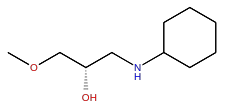 | | 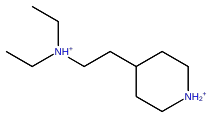 | | 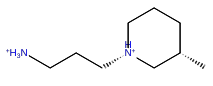 | |
| **(iv) ZINC00285867** | | **(v) ZINC01655611** | | **(vi) ZINC02013388** | |
| 1-Methoxy-3-phenylamino-propan-2-ol | | 4-(2-Diethylaminoethyl) piperidine | | 3-(3-Methyl-piperidin-1-yl)-propylamine | |
| MW = 181.23 | RB = 5 | MW = 186.34 | RB = 5 | MW = 158.28 | RB = 3 |
| tPSA = 41 | HA = 3 | tPSA = 21 | HA = 2 | tPSA = 32 | HA = 2 |
| xlogP = 1.13 | HD = 2 | xlogP = 1.75 | HD = 3 | xlogP = 0.57 | HD = 4 |
| 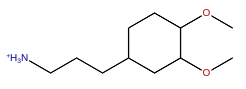 | | 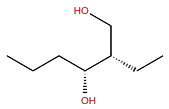 | | 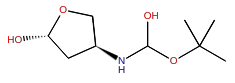 | |
| **(vii) ZINC02981238** | | **(viii) ZINC03860446** | | **(ix) ZINC04202786** | |
| 3-(3,4-Dimethoxy-phenyl)-propylamine | | 2-Ethyl-1,3-hexanediol | | (S)-3-Boc-Amino-g-butyrolactone | |
| MW = 196.27 | RB = 5 | MW = 146.28 | RB = 5 | MW = 201.22 | RB = 3 |
| tPSA = 46 | HA = 3 | tPSA = 40 | HA = 2 | tPSA = 65 | HA = 5 |
| xlogP = 1.09 | HD = 3 | xlogP = 1.72 | HD = 2 | xlogP = 1.77 | HD = 1 |
| 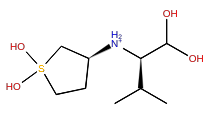 | | 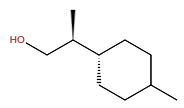 | | 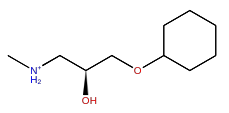 | |
| **(x) ZINC04343210** | | **(xi) ZINC04528592** | | **(xii) ZINC12955012** | |
| N-(1,1-Dioxidotetrahydro-3-thienyl)valine | | (+)-P-menth-1-en-9-ol | | 1-Methylamino-3-phenoxy-propan-2-ol | |
| MW = 235.30 | RB = 4 | MW = 154.25 | RB = 2 | MW = 182.24 | RB = 5 |
| tPSA = 90 | HA = 5 | tPSA = 20 | HA = 1 | tPSA = 46 | HA = 3 |
| xlogP = 2.54 | HD = 2 | xlogP = 2.54 | HD = 1 | xlogP = 1.13 | HD = 3 |
| 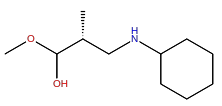 | | 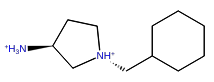 | | 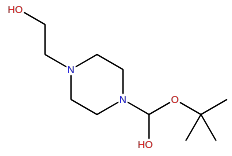 | |
| **(xiii) ZINC13355674** | | **(xiv) ZINC19230174** | | **(xv) ZINC19850539** | |
| methyl 3-anilino-2-methylpropanoate | | 1-Benzylpyrrolidin-3-amine | | tert-butyl 4-(2-hydroxyethyl) piperazine-1-carboxylate | |
| MW = 193.24 | RB = 5 | MW = 178.27 | RB = 2 | MW = 230.30 | RB = 4 |
| tPSA = 38 | HA = 3 | tPSA = 32 | HA = 2 | tPSA = 53 | HA = 5 |
| xlogP = 2.40 | HD = 1 | xlogP = 0.74 | HD = 4 | xlogP = 0.64 | HD = 1 |

**S2 Schema. Fifteen shortlisted ligands as lead compounds for TgFabD**
