# Supplementary material for: Multivariate PLS Modeling of Apicomplexan FabD-Ligand Interaction Space for Mapping Target-Specific Chemical Space and Pharmacophore Fingerprints
Source: PLoS One. 2015 Nov 4;10(11):e0141674. doi: 10.1371/journal.pone.0141674 (PMC4633102; doi:10.1371/journal.pone.0141674)
Supplement: S1 Table — (DOCX) [file pone.0141674.s003.docx]

**S1 Table.** Presenting the active site residues of Apicomplexan and Host FabDs for a relative comparison

| **Active site residues** | **Apicomplexan FabD** | | **Host FabD** | |
| --- | --- | --- | --- | --- |
|  | **PfFabD** | **TgFabD** | **HsFabD** | **EcFabD** |
|  | Gly108 | Gly179 | Gly33 | Gly10 |
|  | Gln109 | Gln180 | Gln34 | Gln11 |
|  | Gly110 | Gly181 | Gly35 | Gly12 |
|  | Gln161 | Gln232 | Thr81 | Thr59 |
|  | Ser193 | Ser267 | Gln85 | Gln63 |
|  | Leu194 | Leu268 | Ser117 | Ser92 |
|  | Gly195 | Gly269 | Gly119 | Gly94 |
|  | Tyr197 | Tyr271 | Arg142 | Arg117 |
| **Identical and occupying same position** | Arg218 | Arg298 | Met146 | Met121 |
|  | Met222 | Met296 | Met157 | Met132 |
|  | Ala303 | Ala397 | Asn193 | Asn160 |
|  | Phe304 | Phe398 | Val201 | Val168 |
|  | His305 | His397 | Leu227 | Leu194 |
|  | Ser306 | Ser400 | His234 | His201 |
|  | Met309 | Met403 | Met238 | Met205 |
|  | Gln354 | Gln448 | Gln283 | Gln250 |
|  | Leu355 | Leu449 | Leu284 | Leu251 |
|  | Thr356 | Thr450 | Val288 | Val255 |
|  | Ile164 | Leu235 | Ile98 | Leu66 |
|  | Tyr112 | Leu266 | Phe116 | His91 |
|  | Thr233 | Met307 | Val117 | Leu93 |
|  | Val262 | Ala345 | Ser192 | Val159 |
| **Substituted but occupying same position** | Ser263 | Asn346 | Gly231 | Val198 |
|  | Ile300 | Val394 | Ala232 | Pro199 |
|  | Ile359 | Val353 | Phe233 | Ser200 |
|  | Ile383 | Val478 | Thr235 | Cys202 |
|  |  |  | Val285 | Tyr252 |
|  |  |  | Ser286 | Asn253 |
|  | Glu111 | Pro178 | Pro9 | Ser203 |
| **Unique and at different positions** | Leu298 | Thr228 | Asn162 | Val229 |
|  | Ser357 | Ser395 |  |  |
|  |  | Met455 |  |  |
