# Supplementary material for: Multivariate PLS Modeling of Apicomplexan FabD-Ligand Interaction Space for Mapping Target-Specific Chemical Space and Pharmacophore Fingerprints
Source: PLoS One. 2015 Nov 4;10(11):e0141674. doi: 10.1371/journal.pone.0141674 (PMC4633102; doi:10.1371/journal.pone.0141674)
Supplement: S2 Table — (DOCX) [file pone.0141674.s004.docx]

**S2 Table.** Details of the amino acids participating in various intermolecular interactions with the shortlisted hits specific to PfFabD

| **Hits** | **Hydrogen Bonds** | | **Hydrophobic** | **Polar** | **Positively charged residues** | **Pi-Cation** |
| --- | --- | --- | --- | --- | --- | --- |
|  | **Main Chain** | **Side Chain** |  |  |  |  |
| ZINC00002170 | - | ARG218 | TYR192, MET222, VAL234, ALA235, MET265, LEU298, PHE304, LEU355, ILE359 | SER193, THR233, SER263, GLN354 | ARG218, LYS296 | - |
| ZINC00164148 | - | ARG218, THR233 | TYR192, MET222, ALA235, VAL262, MET265, LEU298, PHE304, ILE359 | GLN109, SER193, THR233, SER263 | ARG218 | - |
| ZINC00348080 | - | ARG218 | TYR192, MET222, VAL234, ALA235, VAL262, MET265,  LEU298, PHE304, LEU355,  ILE359 | GLN109, SER193, THR233, SER263, HIS305, GLN354 | ARG218, LYS296, LYS297 | - |
| ZINC00873422 | - | ARG218 | TYR192, MET222, ALA235, VAL262, MET265, PHE304, ILE359, LEU355 | GLN109, SER193, THR233, SER263, HIS305, GLN354 | ARG218 | - |
| ZINC01529532 | - | ARG218 | TYR192, LEU194, ALA235, VAL262, MET265, LEU298, PHE304, ILE359 | GLN109, SER193, THR233, SER263 | ARG218 | - |
| ZINC01688939 | - | ARG218 | TYR192, LEU194, MET222, VAL262, MET265, LEU298, PHE304, ILE359 | GLN109, SER193, THR233, SER263 | ARG218 | - |
| ZINC02386282 | - | GLN109, THR233 | TYR192, MET222, ALA235, VAL262, MET265, LEU298, PHE304, ILE359, LEU355,  ILE361 | GLN109, SER193, THR233, SER263, HIS305, GLN354 | ARG218 | - |
| ZINC03705320 | GLN109 | - | TYR192, LEU194, MET222, VAL262, MET265, LEU298,  ILE300, ALA301, PHE304,  ILE359 | GLN109, SER157, GLN161, SER263, THR233 | ARG218 | PHE304 |
| ZINC04899687 | - | ARG218 (2) | TYR192, MET222, VAL262, MET265, LEU298, PHE304,  ILE359, ILE361 | GLN109, SER193, THR233, SER263 | ARG218 | PHE304 |
| ZINC05234667 | - | GLN109 (2) | TYR192, MET222, VAL262, MET265, PHE304, LEU355,  ILE359 | GLN109, SER193, SER263, HIS305, SER357 | ARG218 | - |
| ZINC13378724 | GLN109 | TYR192 | TYR192, LEU194, MET222, VAL262, MET265, LEU298, PHE304, ILE359 | GLN109, SER193, THR233, SER263 | ARG218 | - |
| ZINC13413550 | GLN109 (2) | SER263 | TYR192, MET222, ALA235, VAL262, MET265, LEU298, PHE304, ILE359 | GLN109, SER193, THR233, SER263 | ARG218 | - |
| ZINC13435849 | GLN354 | ARG218 (2) | TYR192, MET222, ALA235, VAL262, MET265, LEU298, PHE304, LEU355, ILE359 | GLN109, SER193, THR233, SER263, HIS305, GLN354 | ARG218 | - |
| ZINC20357842 | - | TYR192, SER263, ARG218 | TYR192, VAL262, MET265, LEU298, MET222, PHE304,  ILE359 | GLN109, SER193, STHR233, SER263, SER157, GLN161 | ARG218 | - |
| ZINC20357942 | - | SER263 | TYR192, VAL262, PHE304, LEU298, MET222, ILE300, MET265, ILE359 | GLN109, SER157, SER193, SER263, THR233 | ARG218 | - |
