# Supplementary material for: Multivariate PLS Modeling of Apicomplexan FabD-Ligand Interaction Space for Mapping Target-Specific Chemical Space and Pharmacophore Fingerprints
Source: PLoS One. 2015 Nov 4;10(11):e0141674. doi: 10.1371/journal.pone.0141674 (PMC4633102; doi:10.1371/journal.pone.0141674)
Supplement: S3 Table — (DOCX) [file pone.0141674.s005.docx]

**S3 Table.** Details of the amino acids participating in various intermolecular interactions with the shortlisted hits specific to TgFabD

| **Hits** | **Hydrogen Bonds** | | **Hydrophobic** | **Polar** | **Positively charged residues** | **Pi-Cation** |
| --- | --- | --- | --- | --- | --- | --- |
|  | **Main Chain** | **Side Chain** |  |  |  |  |
| ZINC00002159 | GLY476, GLY477 | - | PRO178, ALA182, MET187, LEU235, LEU266, VAL478, PRO475 | GLN180, GLN183 | - | - |
| ZINC00154890 | GLN180, LEU266, HIS399 | ASN346 | LEU266, LEU268, MET307, ALA342, LEU348, VAL354, LEU392, PHE398, LEU482, VAL478 | GLN180, SER267, ASN346, HIS399 | - | - |
| ZINC00226411 | HIS399 | - | LEU266, MET307, LEU348, ALA345, VAL354, LEU392, PHE398, VAL478, | GLN180, SER267, ASN346, HIS399 | - | - |
| ZINC00285867 | - | ARG292 | LEU266, LEU268, MET296, LEU268, MET307, LEU348, VAL354, PHE398, VAL478 | GLN180, GLN232, SER267, ASN346, HIS3999 | ARG292 | - |
| ZINC01655611 | - | - | ALA182, LEU266, LEU268, MET307, LEU348, VAL354, LEU392, VAL394, PHE398, VAL478 | GLN180, SER267, ASN346, HIS399 | - | - |
| ZINC02013388 | GLN180 | SER267 | LEU266, LEU268, MET307, LEU348, VAL354, LEU392, PHE398, LEU482, VAL478 | GLN180, SER267, ASN346, HIS399 | ARG292 | - |
| ZINC02981238 | GLY179, ALA182 | - | ALA182, PRO178, LEU266, LEU235, LEU348, PRO475, VAL478 | GLN180, GLN183 | - | - |
| ZINC03860446 | GLN180 | - | LEU266, LEU268, MEY307, LEU348, VAL354, LEU392, PHE398, VAL478, LEU482 | GLN180, SER267, ASN346, HIS399 | - | - |
| ZINC04202786 | - | - | ALA182, LEU266, PHE398, LEU348, MET307, VAL354, VAL478, LEU482, VAL394, LEU392, | GLN180, ASN346 | - | - |
| ZINC04343210 | GLN180 (2) |  | ALA182, LEU266, MET307, LEU348, VAL354, LEU392, PHE398, VAL478, LEU482 | GLN180, SER267, ASN346, HIS399 | - |  |
| ZINC04528592 | HIS399 | ASN346 | ALA182, LEU266, LEU268, LEU348, VAL354, LEU392, PHE398, VAL478 | GLN180, SER267, ASN3436, HIS399 |  | - |
| ZINC12955012 | HIS399 | - | LEU266, LEU268, MET307, LEU348, VAL354, LEU392, PHE398, VAL478 | GLN180, SER267, ASN346, HIS399 | ARG292 | - |
| ZINC13355674 | - | - | ALA182, LEU266, MET307, LEU348, VAL354,LEU393, VAL394, PHE398,VAL478, LEU482 | GLN180, SER267, ASN3436, HIS399 | - | - |
| ZINC19230174 | GLN180 (2) | - | ALA182, LEU266, LEU268, PHE298, MET307, LEU348, VAL354, LEU392, VAL394, VAL478, | GLN180, SER267, ASN3436, HIS399 | - | - |
