# Supplementary material for: Multivariate PLS Modeling of Apicomplexan FabD-Ligand Interaction Space for Mapping Target-Specific Chemical Space and Pharmacophore Fingerprints
Source: PLoS One. 2015 Nov 4;10(11):e0141674. doi: 10.1371/journal.pone.0141674 (PMC4633102; doi:10.1371/journal.pone.0141674)
Supplement: S4 Table — (DOCX) [file pone.0141674.s006.docx]

**S4 Table.** Inter-molecular interactions of HsFabD with the shortlisted leads of PfFabD

| **Ligands** | **Hydrogen bonds** | | **Hydrophobic Interactions** | **Polar Interactions** | **Pi-Cation** |
| --- | --- | --- | --- | --- | --- |
|  | **Main –Chain** | **Side-chain** |  |  |  |
| ZINC00002170 |  | HIS234 | PHE116, VAL201, LEU195, ILE321, VAL229, MET157, LEU227, PHE233 | GLN34, SER36, SER159, GLN317, ASN193, HIS234, SER117 |  |
| ZINC00164148 | GLY33 |  | PRO32, PHE116, LEU318, ILE321, LEU195, VAL201, MET157, VAL229, LEU227, PHE233 | GLN34, SER36, GLN317, ASN193, HIS234 |  |
| ZINC00348080 | GLN34 | SER159 | PHE116, VAL201, MET157, LEU195, PHE196, CYC199, ILE321, PHE233, VAL229 | GLN34, SER117, HIS234, ASN193, SER159, GLN317 |  |
| ZINC00873422 |  |  | PHE116, PRO228, LEU227, VAL229, MET157, PHE233 | GLN34, ASN193, GLN317 |  |
| ZINC01529532 | GLY33 | SER36 | PRO32, LEU195, ILE321, LEU227, PHE116, LEU318 | GLN34, SER36, SER159, GLN317 |  |
| ZINC01688939 |  |  | PHE116, LEU318, ILE321, LEU227, LEU195, VAL201 | GLN34, SER36, ASN193, SER159, GLN317 |  |
| ZINC02386282 | GLN34 | GLN317 | PHE116, LEU227, LEU195, ALA320, ILE321 | GLN34, SER36, GLN317, ASN193, HIS234 |  |
| ZINC03705320 |  |  | PHE116, LEU227, VAL201, LEU195, PHE196, ILE321, ALA320 | GLN34, SER159, GLN317 |  |
| ZINC04899687 | GLY33 | GLN317 | PHE116, LEU318, PRO32, ILE321, LEU195, VAL201, LEU227 | GLN34, SER36, GLN317 | PHE116 |
| ZINC05234667 |  | GLN317 | PHE116, VAL201, LEU195, LEU227 | GLN34, SER36, SER159, GLN317 |  |
| ZINC13378724 |  |  | PHE116, LEU318, LEU192, ILE321, VAL201, LEU227 | GLN34, SER36, SER159, GLN317 |  |
| ZINC13413550 |  |  | PHE116, LEU195, ILE321, LEU227, PRO228, VAL229, PHE233, MET157, VAL201, LEU318 | GLN34, SER36, SER159, ASN193, HIS234, GLN317 |  |
| ZINC13435849 |  |  | PHE116, LEU195, LEU227, MET226, PRO228, VAL229, MET157, PHE233, VAL201 | GLN34, ASN193, HIS234, GLN317 |  |
| ZINC20357842 |  |  | PHE116, VAL201, MET157, LEU227, LEU195, ILE321 | GLN34, ASN193, SER157, HIS234, GLN317 |  |
| ZINC20357942 |  | SER159 | PHE116, ILE321, LEU227, LEU195, VAL201, LEU318 | GLN34, SER36, SER159, GLN317 |  |
