# Supplementary material for: Multivariate PLS Modeling of Apicomplexan FabD-Ligand Interaction Space for Mapping Target-Specific Chemical Space and Pharmacophore Fingerprints
Source: PLoS One. 2015 Nov 4;10(11):e0141674. doi: 10.1371/journal.pone.0141674 (PMC4633102; doi:10.1371/journal.pone.0141674)
Supplement: S5 Table — (DOCX) [file pone.0141674.s007.docx]

**S5 Table.** Inter-molecular interactions of HsFabD with the shortlisted leads of TgFabD

| **Ligands** | **Hydrogen bonds** | | **Hydrophobic Interactions** | **Polar Interactions** | **Pi-Cation** |
| --- | --- | --- | --- | --- | --- |
|  | **Main –Chain** | **Side-chain** |  |  |  |
| ZINC00002159 | GLN34 |  | PRO228, VAL229, LEU227, ILE321, LEU195, PHE116, LEU318 | GLN34, SER36, GLN317 |  |
| ZINC00154890 |  |  | PHE116, ILE321, ILE195, VAL201, PHE233, MET157, PRO228, LEU227, VAL229 | GLN34, SER159, ASN193, HIS234, GLN317 |  |
| ZINC00226411 |  |  | PHE116, LEU318, LEU227, MET157, VAL229, PHE233, MET146, VAL201, LEU195, ILE321, PRO32 | GLN34, SER36, SER159, ASN193, SER117, HIS234, GLN317 |  |
| ZINC00285867 |  |  | VAL201, PHE116, LEU318, PRO32, LEU195, ILE321, LEU227, VAL229, PHE233, MET157 | GLN34, SER36, ASN193, HIS234, GLN317 |  |
| ZINC01655611 |  |  | LEU318, PHE116, ILE321, LEU195, VAL201, MET157, PHE233, VAL229, LEU227 | GLN34, SER36, ASN193, HIS234, GLN317 |  |
| ZINC02013388 |  |  | LEU318, PHE116, LEU227, VAL201, VAL229, PHE233, MET157, ILE321, ILE195 | GLN34, SER36, ASN193, HIS234, GLN317 |  |
| ZINC02981238 | GLN34 |  | PRO228, LEU227, PHE116, VAL201, VAL229, PHE233, MET157 | GLN34, SER159, HIS234, GLN317 |  |
| ZINC03860446 |  |  | LEU227, MET157, VAL201, ILE321, LEU195, LEU318, PHE116, PRO32 | GLN34, SER36, SER159, ASN193, GLN317 |  |
| ZINC04202786 |  |  | LEU227, VAL201, LEU195, PHE116, LEU318, ILE321 | GLN34, SER36, SER159, GLN317 |  |
| ZINC04343210 |  | SER159 | PHE196, CYS199, ALA320, LEU195, ILE321, LEU227 | SER159, GLN317 |  |
| ZINC04528592 |  |  | LEU195, ILE321, LEU318, PRO32, PHE116, LEU227, VAL201 | GLN34, SER36, SER159, GLN317 |  |
| ZINC12955012 |  | GLN34, SER117, HIS234 | PHE116, VAL201, LEU195, LEU227, MET157, PHE233, MET146, VAL229 | GLN34, THR81, GLN85, SER117, ASN193, HIS234 | PHE233 |
| ZINC13355674 |  |  | PHE116, PRO32, ILE321, LEU318, LEU195, VAL201, LEU227, VAL229, PHE233, MET157 | GLN34, SER36, ASN193, HIS234, GLN317 |  |
| ZINC19230174 | GLY33 | GLN317 | PHE116, PRO32, LEU318, ILE321, LEU195, VAL201, PRO228, LEU227, VAL229 | GLN34, SER36, GLN317 | PHE116 |
| ZINC19850539 |  | SER36 | PHE116, LEU318, PRO32, LEU195, ILE321, VAL201, LEU227, PRO228 | GLN34, SER36, SER159, GLN317 |  |
