# Supplementary material for: Multivariate PLS Modeling of Apicomplexan FabD-Ligand Interaction Space for Mapping Target-Specific Chemical Space and Pharmacophore Fingerprints
Source: PLoS One. 2015 Nov 4;10(11):e0141674. doi: 10.1371/journal.pone.0141674 (PMC4633102; doi:10.1371/journal.pone.0141674)
Supplement: S6 Table — (DOCX) [file pone.0141674.s008.docx]

**S6 Table.** List of structural and physiochemical ligand descriptors contributing positively towards binding affinity of different PLS models

| **Response Variables** | **All-FabDs** | **Pathogen-FabDs** | **Apicomplexan-FabDs** | **Host-FabDs** | **PfFabD** | **TgFabD** | **HsFabD** |
| --- | --- | --- | --- | --- | --- | --- | --- |
| **dG-Amber** | AMR, MLOGP, nArCONHR, nAT, nBT, nCIR, nCrs, nCs, nH, nImidazole, nPyrroles, nR05, nR06, nR09, nR10, nRCOOH, nRN, nROH, nS, Se, Sp, WI, nR07 | AMR, MLOGP, nArCONHR, nAT, nBT, nCconj, nCrs, nCrt, nCs, nH, nHAcc, nPyridines, nPyrroles, nPyrrolidines, nR05, nR06, nR10, nRCOOH, nCIC, nROH, nS, RBF, RBN, Se, Sp, Sv, WI | AMR, MLOGP, Ms, nArCONHR, nAT, nBT, nCb-, nCconj, nCrs, nCrt, nCs, nCt, nDB, nH, nHAcc, nO, nPyrroles, nPyrrolidines, nR06, nR09, nRCOOH, nROH, nS, RBN, Se, WI | - | Me, AMR, MLOGP , Ms, nArCONH2, nArCONHR, nArCOOH, nBnz, nCb-, nCbH, nCconj, nCIR, nCrs, nCrt, nCs, nCt, nDB, nHAcc, nO, nCIC, nPyrroles, nPyrrolidines, nR=Cs, nR05, nR07, nR09, nR10, nRCOOH, nROH, nS, WI | AMR, MW, MLOGP , nArCONHR, nAT, nC, nCp, nCrs, nCrt, nCs, nCt, nDB, nH, nHAcc, nNq, nO, nBT, nSK, nBO, nPyrrolidines, nR06, nRCONR2, nRCOOH, nROH, nS, RBF, RBN, Se, Sp, Sv, WI | - |
| **ElecStat** | AMW, Me, Mp, Ms, Mv, nArCONH2, nArCOOH, nBnz, nCb-, nCbH, nCconj, nDB, nHDon, nO, nPyrroles, nR=Cs, nR09, nROH, nS | AMW, ARR, Me, Mp, Ms, Mv, nArCONH2, nArCOOH, nBM, nBnz, nCar, nCb-, nCbH, nCconj, nCIR, nCj, nDB, nHDon, nO, nPyridines, nPyrroles, nR=Cs, nR06, nR09, nR10, nRN, nROH, nS, RBF | AMW, AMR, Me, MLOGP, Mp, Ms, Mv, nArCONH2, nArCONHR, nArCOOH, nBnz, nCar, nCb-, nCbH, nCconj, nCIR, nCrs, nCrt, nDB, nHAcc, nO, nPyrroles, nPyrrolidi, nR06, nR09, nRCOOH, nROH, nS, RBF, WI | AMR, Mp, Ms, Mv, nArCONH2, nArCONHR, nArCOOH, nBnz, nC, nCar, nCb-, nCbH, nCconj, nCIR, nCrs, nCrt, nCt, nDB, nO, nPyridines, nPyrroles, nR=Cs, nR06, nR07, nR09, nR10, nRCOOH, nROH, nS, RBF | AMW, Me, molar refr, Moriguchi , Mp, Ms, Mv, nArCONH2, nArCONHR, nArCOOH, nBnz, nCb-, nCbH, nCconj, nCIR, nCrs, nCs, nDB, nHAcc, nO, nPyrroles, nPyrrolidi, nR=Cs, nR06, nR09, nR10, nRCOOH, nROH, nS, WI | AMW, ARR, Me, AMR, MLOGP, Mp, Ms, Mv, nArCONH2, nArCOOH, nBnz, nCar, nCb-, nCbH, nCIR, nCrt, nHDon, nImidazole, nAB, nBM, nCIC, nPyridines, nPyrroles, nR06, nR09, nRCONR2, nROH, nS, RBF, SCBO | AMR, Hy, Mp, Ms, Mv, nArCONHR, nArCOOH, nBnz, nCb-, nCbH, nCconj, nCIR, nCrt, nCt, nDB, nHDon, nO, nPyridines, nPyrroles, nPyrrolidi, nR=Cs, nR06, nR09, nR10, nRCOOH, nROH, nS |
| **VDW** | AMR, AMW, Me, MLOGP, MW, nArCONHR, nAT, nBM, nBO, nBT, nC, nCar, nCj, nCrs, nCrt, nCs, nDB, nH, nHAcc, nImidazole, nN, nO, nPyridines, nPyrrolidi, nR05, nR06, nR10, nRCONHR, nRCONR2, nRCOOH, nRN, nSK, RBF, RBN, SCBO, Se, Sp, Ss, Sv, TPSA, Wi | AMR, MW, nArCONHR, nAT, nBM, nBO, nBT, nC, nCar, nCconj, nCj, nCrs, nCs, nH, nHAcc, nImidazole, nN, nPyridines, nR06, nR10, nRCONHR, nRN, nROH, nSK, RBF, RBN, SCBO, Se, Sp, Ss, Sv, TPSA, WI | MW, nArCONHR, nAT, nBO, nBT, nC, nCar, nCconj, nCj, nCrs, nCs, nH, nHAcc, nHDon, nImidazole, nN, nPyridines, nPyrrolidi, nR06, nR10, nRCONHR, nRCOOH, nRN, nROH, nSK, RBF, RBN, SCBO, Se, Sp, Ss, Sv, TPSA, WI | AMW, Hy, Me, MLOGP, MW, nArCONHR, nAT, nBO, nBT, nC, nCIR, nCp, nCrs, nCrt, nCs, nDB, nH, nHAcc, nHBonds, nHDon, nImidazole, nN, nN+, nNq, nO, nR05, nR07, nRCONHR, nRCONR2, nRN, nSK, RBF, RBN, SCBO, Se, Sp, Ss, Sv, TPSA, WI | MW, AMW, Me, AMR, MLOGP, Ms, nArCONHR, nAT, nC, nCIR, nCp, nCrs, nCrt, nCs, nCt, nDB, nH, nHAcc, nHBonds, nImidazole, nN, nN+, nNq, nO, nPyridines, nR05, nR06, nR07, nR10, nRCONHR, nRCONR2, nRCOOH, nROH, RBF, RBN, SCBO, Se, Sp, Sv, TPSA,WI | ARR, Hy, nArCONHR, nAT, nBnz, nC, nCar, nCb-, nCbH, nCconj, nCs, nH, nHDon, nImidazole, nN, nN+, nNq, nBT, nAB, nBM, nSK, nBO, nPyridines, nPyrrolidi, nR=Cs, nR06, nRCONHR, RBF, RBN, Se, Sp, Sv | AMW, Me, MLOGP, Ms, MW, nArCONHR, nAT, nC, nCp, nCrs, nCrt, nCs, nCt, nDB, nH, nHAcc, nHBonds, nImidazole, nN, nN+, nNq, nO, nBT, nBM, nSK, nCIC, nBO, nPyridines, nPyrrolidines, nR05, nR07, nR10, nRCONHR, nRCONR2, nRCOOH, RBF, RBN, SCBO, Se, Sp, Sv, TPSA, WI |
