# Supplementary material for: Multivariate PLS Modeling of Apicomplexan FabD-Ligand Interaction Space for Mapping Target-Specific Chemical Space and Pharmacophore Fingerprints
Source: PLoS One. 2015 Nov 4;10(11):e0141674. doi: 10.1371/journal.pone.0141674 (PMC4633102; doi:10.1371/journal.pone.0141674)
Supplement: S7 Table — (DOCX) [file pone.0141674.s009.docx]

**S7 Table.** List of protein z-scale descriptors contributing positively towards binding affinity of different PLS models

| **Response Variables** | **All-FabDs** | **Pathogen-FabDs** | **Apicomplexan-FabDs** | **Host-FabDs** |
| --- | --- | --- | --- | --- |
| **dG-Amber** | 157_z1, 157_z2, 157_z3, 157_z4, 157_z5, 194_z2, 194_z3, 194_z4, 194_z5, 228_z1, 228_z2, 228_z3, 228_z5, 229_z2, 229_z3, 229_z4, 231_z2, 231_z4, 232_z4, 232_z5, 233_z1, 233_z5, 235_z4, 235_z5, 263_z3, 265_z3, 265_z4, 271_z1, 271_z3, 271_z4, 296_z5, 297_z1, 297_z2, 297_z4, 297_z5, 299_z1, 299_z2, 300_z2, 300_z4, 300_z5, 301_z4, 301_z5, 302_z2, 302_z5, 303_z1, 304_z1, 304_z3, 304_z5, 359_z2, 359_z4, 359_z5 | 157_z1, 157_z2, 157_z3, 157_z4, 157_z5, 192_z2, 192_z3, 228_z2, 228_z3, 228_z5, 229_z2, 229_z3, 229_z4, 231_z2, 231_z3, 231_z4, 232_z4, 233_z1, 233_z5, 263_z3, 265_z4, 271_z1, 271_z3, 271_z4, 296_z1, 296_z5, 297_z1, 297_z2, 297_z4, 299_z1, 299_z2, 299_z3, 300_z2, 300_z4, 300_z5, 301_z4, 301_z5, 302_z2, 302_z5, 303_z1, 304_z1, 304_z3, 304_z5, 359_z2, 359_z4, 359_z5 | 157_z1, 157_z2, 157_z3, 157_z4, 157_z5, 192_z1, 192_z2, 192_z3, 192_z4, 228_z4, 228_z5, 229_z2, 229_z4, 231_z2, 231_z3, 231_z4, 232_z2, 233_z1, 233_z5, 263_z3, 265_z1, 265_z2, 265_z3, 265_z4, 271_z1, 271_z3, 271_z4, 296_z1, 296_z2, 296_z4, 296_z5, 297_z3, 297_z5, 299_z1, 299_z3, 300_z2, 300_z4, 300_z5, 301_z4, 301_z5, 303_z2, 303_z3, 303_z4, 303_z5, 359_z2, 359_z4, 359_z5 | - |
| **ElecStat** | 157_z1, 157_z2, 157_z3, 157_z4, 157_z5, 192_z1, 192_z2, 192_z3, 192_z4, 194_z1, 228_z4, 229_z4, 231_z2, 231_z3, 231_z4, 231_z5, 232_z1, 232_z2, 232_z3, 232_z5, 233_z1, 233_z5, 235_z1, 235_z2, 235_z3, 262_z1, 262_z2, 262_z3, 262_z5, 263_z3, 265_z1, 265_z2, 265_z3, 265_z5, 271_z1, 271_z3, 271_z4, 296_z1, 296_z2, 296_z3, 296_z4, 297_z3, 297_z5, 299_z3, 299_z5, 300_z2, 300_z4, 300_z5, 301_z4, 301_z5, 302_z1, 302_z3, 302_z4, 303_z2, 303_z3, 303_z4, 303_z5, 304_z2, 304_z4, 359_z2, 359_z4, 359_z5 | 157_z1, 157_z2, 157_z3, 157_z4, 157_z5, 192_z1, 192_z2, 192_z3, 192_z4, 228_z4, 229_z4, 231_z2, 231_z3, 231_z4, 232_z1, 232_z2, 232_z3, 232_z5, 233_z1, 233_z5, 262_z1, 262_z2, 262_z3, 262_z4, 262_z5, 263_z3, 265_z1, 265_z2, 265_z3, 265_z4, 265_z5, 271_z1, 271_z3, 271_z4, 296_z1, 296_z2, 296_z3, 296_z4, 297_z3, 297_z5, 299_z3, 299_z5, 300_z2, 300_z4, 300_z5, 301_z4, 301_z5, 302_z1, 302_z3, 302_z4, 303_z2, 303_z3, 303_z4, 303_z5, 304_z2, 304_z4, 359_z2, 359_z4, 359_z5 | 157_z1, 157_z2, 157_z3, 157_z4, 157_z5, 192_z1, 192_z2, 192_z3, 192_z4, 228_z4, 228_z5, 229_z2, 229_z4, 231_z2, 231_z3, 231_z4, 232_z2, 233_z1, 233_z5, 263_z3, 265_z1, 265_z2, 265_z3, 265_z4, 271_z1, 271_z3, 271_z4, 296_z1, 296_z2, 296_z4, 296_z5, 297_z3, 297_z5, 299_z1, 299_z3, 300_z2, 300_z4, 300_z5, 301_z4, 301_z5, 303_z1, 359_z2, 359_z4, 359_z5 | 192_z3, 194_z1, 228_z3, 228_z4, 228_z5, 229_z2, 229_z5, 231_z1, 231_z2, 231_z3, 231_z5, 232_z1, 235_z1, 235_z2, 235_z3, 262_z1, 262_z2, 262_z3, 262_z5, 265_z4, 296_z1, 296_z2, 296_z4, 297_z4, 302_z1, 302_z3, 302_z4, 303_z1, 304_z2, 304_z4 |
| **VDW** | 157_z1, 157_z2, 157_z3, 157_z4, 157_z5, 192_z2, 192_z3, 194_z1, 228_z1, 228_z2, 228_z3, 228_z5, 229_z2, 229_z4, 231_z2, 231_z3, 231_z4, 231_z5, 232_z4, 232_z5, 233_z1, 233_z5, 235_z1, 235_z2, 235_z3, 263_z3, 265_z4, 271_z1, 271_z3, 271_z4, 296_z1, 296_z2, 296_z5, 297_z3, 297_z4, 299_z2, 299_z3, 299_z4, 299_z5, 300_z2, 300_z4, 300_z5, 301_z4, 301_z5, 302_z2, 302_z5, 303_z1, 304_z1, 304_z3, 304_z5, 359_z2, 359_z4, 359_z5 | 157_z1, 157_z2, 157_z3, 157_z4, 157_z5, 228_z1, 228_z2, 228_z3, 228_z5, 229_z2, 229_z3, 229_z4, 231_z2, 231_z3, 231_z4, 232_z4, 232_z5, 233_z1, 233_z5, 263_z3, 265_z4, 271_z1, 271_z3, 271_z4, 296_z1, 296_z5, 297_z1, 297_z2, 297_z4, 299_z2, 299_z3, 300_z2, 300_z4, 300_z5, 301_z4, 301_z5, 302_z2, 302_z5, 303_z1, 304_z1, 304_z3, 304_z5, 359_z2, 359_z4, 359_z5 | 192_z1, 192_z2, 192_z4, 192_z5, 194_z2, 194_z3, 194_z4, 194_z5, 228_z1, 228_z2, 229_z1, 229_z3, 229_z4, 231_z4, 232_z2, 232_z3, 232_z4, 232_z5, 235_z4, 235_z5, 262_z4, 265_z1, 265_z2, 265_z3, 265_z5, 296_z3, 296_z5, 297_z1, 297_z2, 297_z3, 297_z5, 302_z2, 302_z5, 303_z2, 303_z3, 303_z4, 303_z5, 304_z1, 304_z3, 304_z5 | 157_z1, 157_z2, 157_z3, 157_z4, 157_z5, 192_z1, 192_z2, 192_z3, 192_z4, 228_z4, 228_z5, 229_z2, 229_z4, 231_z2, 231_z3, 231_z4, 232_z2, 232_z5, 233_z1, 233_z5, 263_z3, 265_z1, 265_z2, 265_z3, 265_z4, 271_z1, 271_z3, 271_z4, 296_z1, 296_z2, 296_z4, 296_z5, 297_z3, 297_z5, 299_z1, 299_z3, 300_z2, 300_z4, 300_z5, 301_z4, 301_z5, 303_z2, 303_z3, 303_z4, 303_z5, 359_z2, 359_z4, 359_z5 |
