# Supplementary figures and images for: Multivariate PLS Modeling of Apicomplexan FabD-Ligand Interaction Space for Mapping Target-Specific Chemical Space and Pharmacophore Fingerprints
Source: PLoS One. 2015 Nov 4;10(11):e0141674. doi: 10.1371/journal.pone.0141674 (PMC4633102; doi:10.1371/journal.pone.0141674)

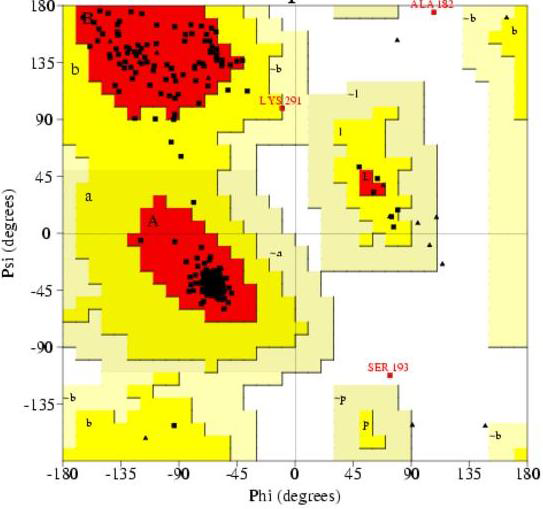

Supplement: S1 Fig — (TIF) [file pone.0141674.s010.tif]

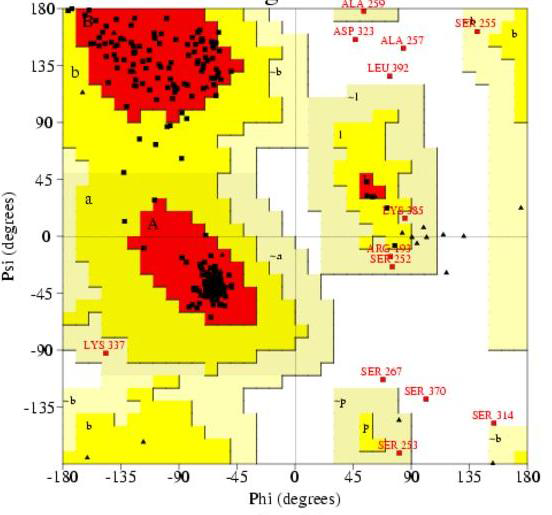

Supplement: S2 Fig — (TIF) [file pone.0141674.s011.tif]

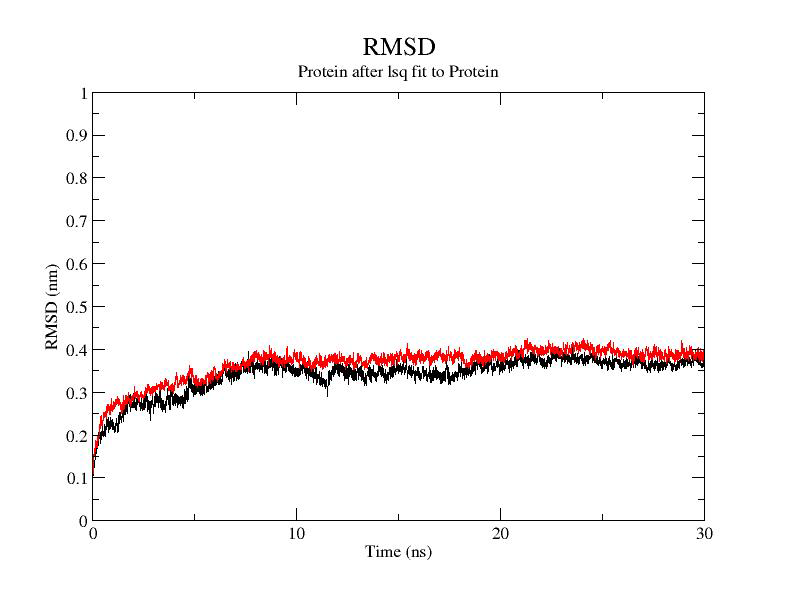

Supplement: S3 Fig — (TIF) [file pone.0141674.s012.tif]

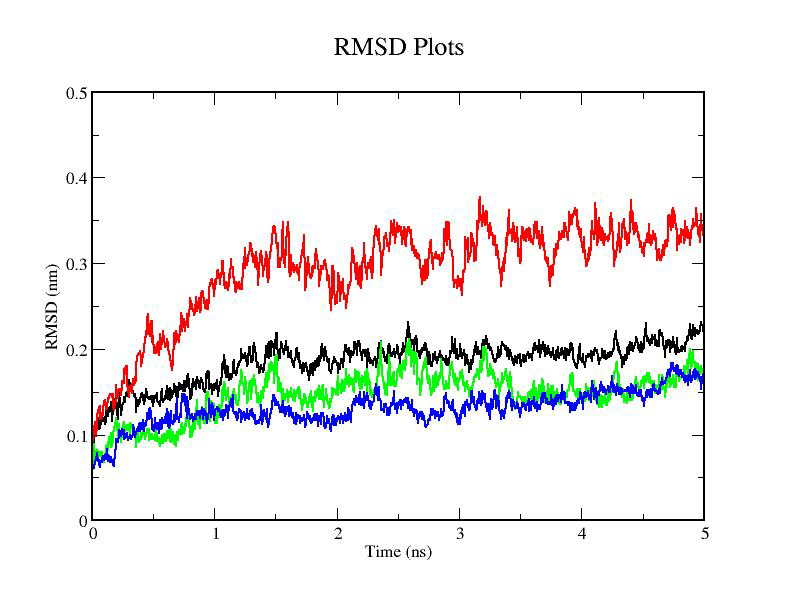

Supplement: S4 Fig — (i) the Cα-backbone coordinates represented in black and red colors, respectively, and (ii) binding residues spanning 6Å region around malonate in its binding pocket represented in green and blue colors, respectively. (TIF) [file pone.0141674.s013.tif]

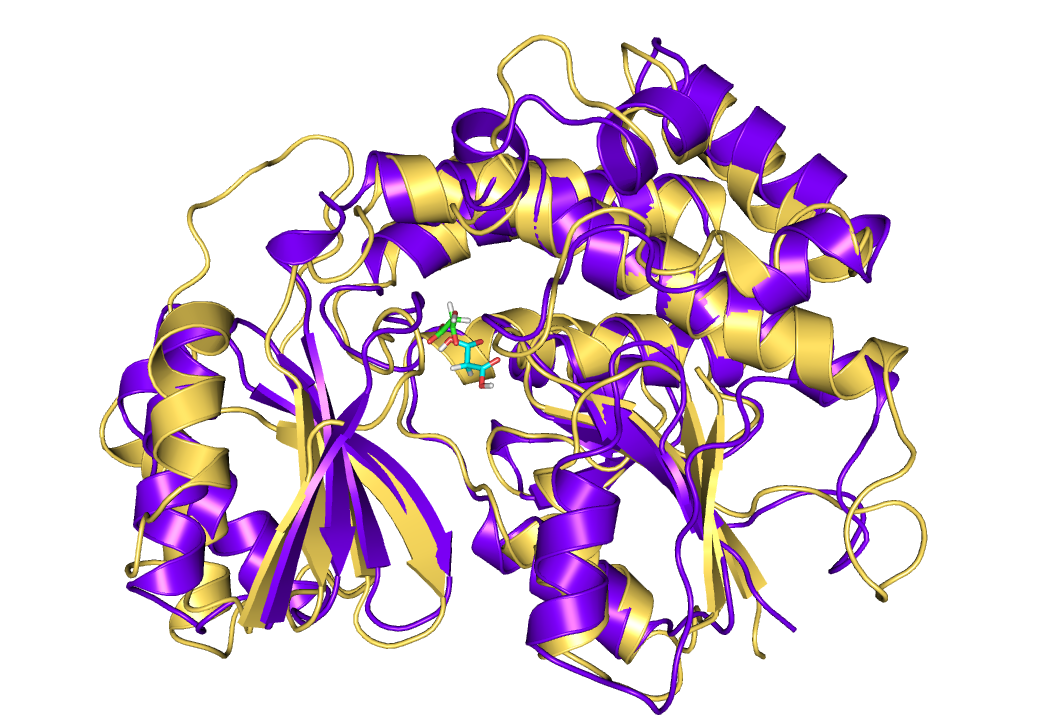

Supplement: S5 Fig — (TIF) [file pone.0141674.s014.tif]

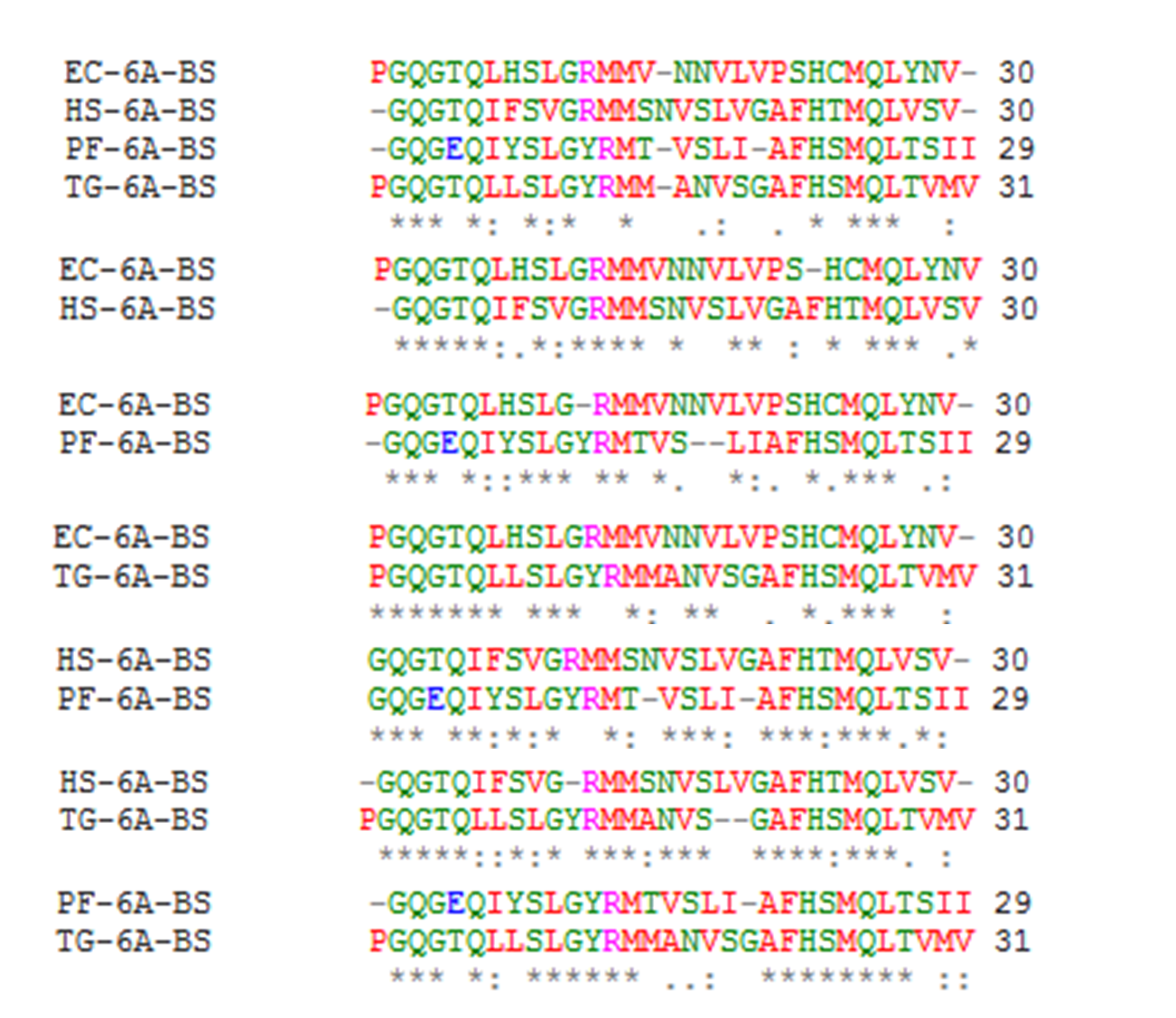

Supplement: S6 Fig — (TIF) [file pone.0141674.s015.tif]

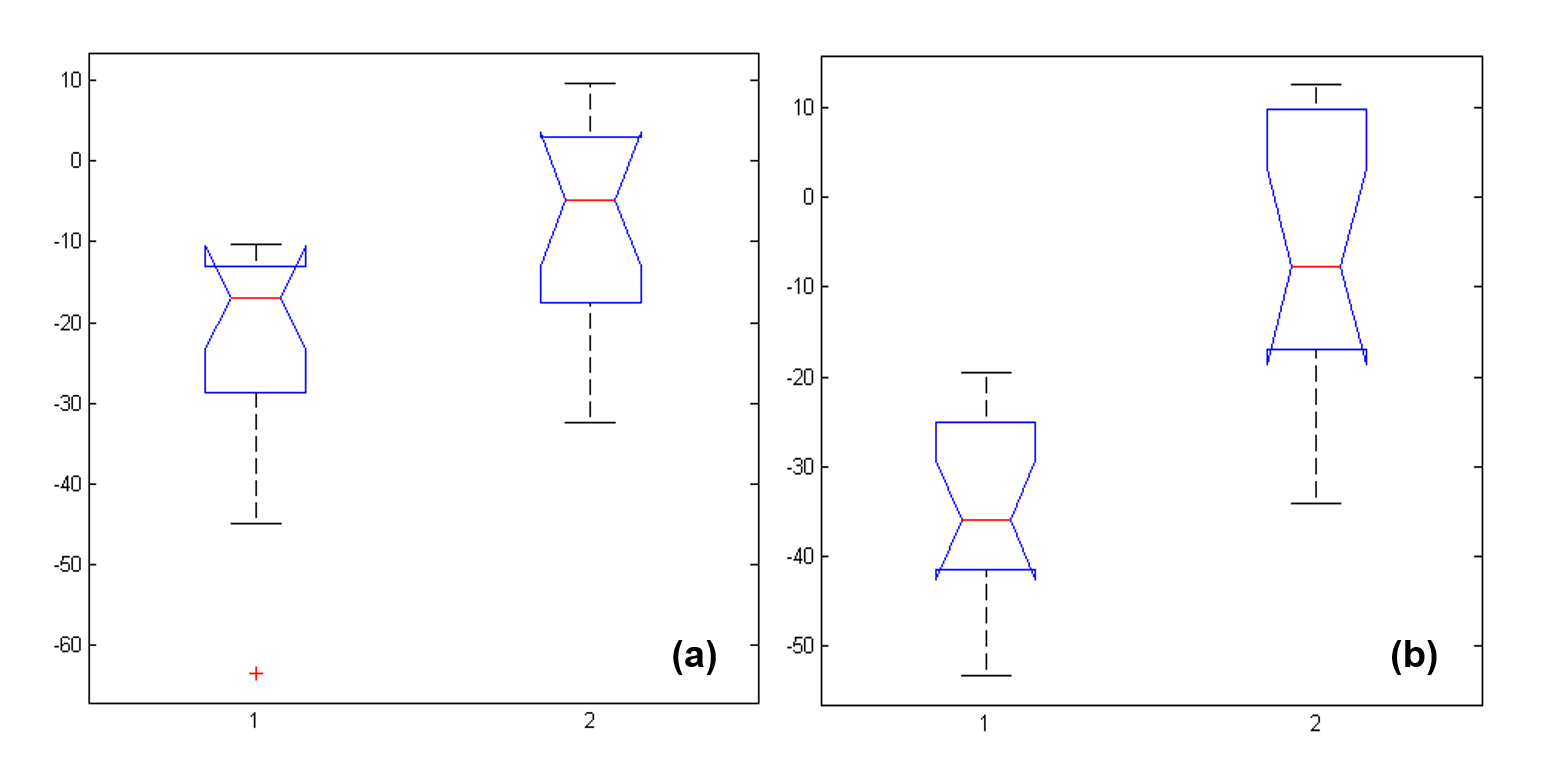

Supplement: S7 Fig — (TIF) [file pone.0141674.s016.tif]

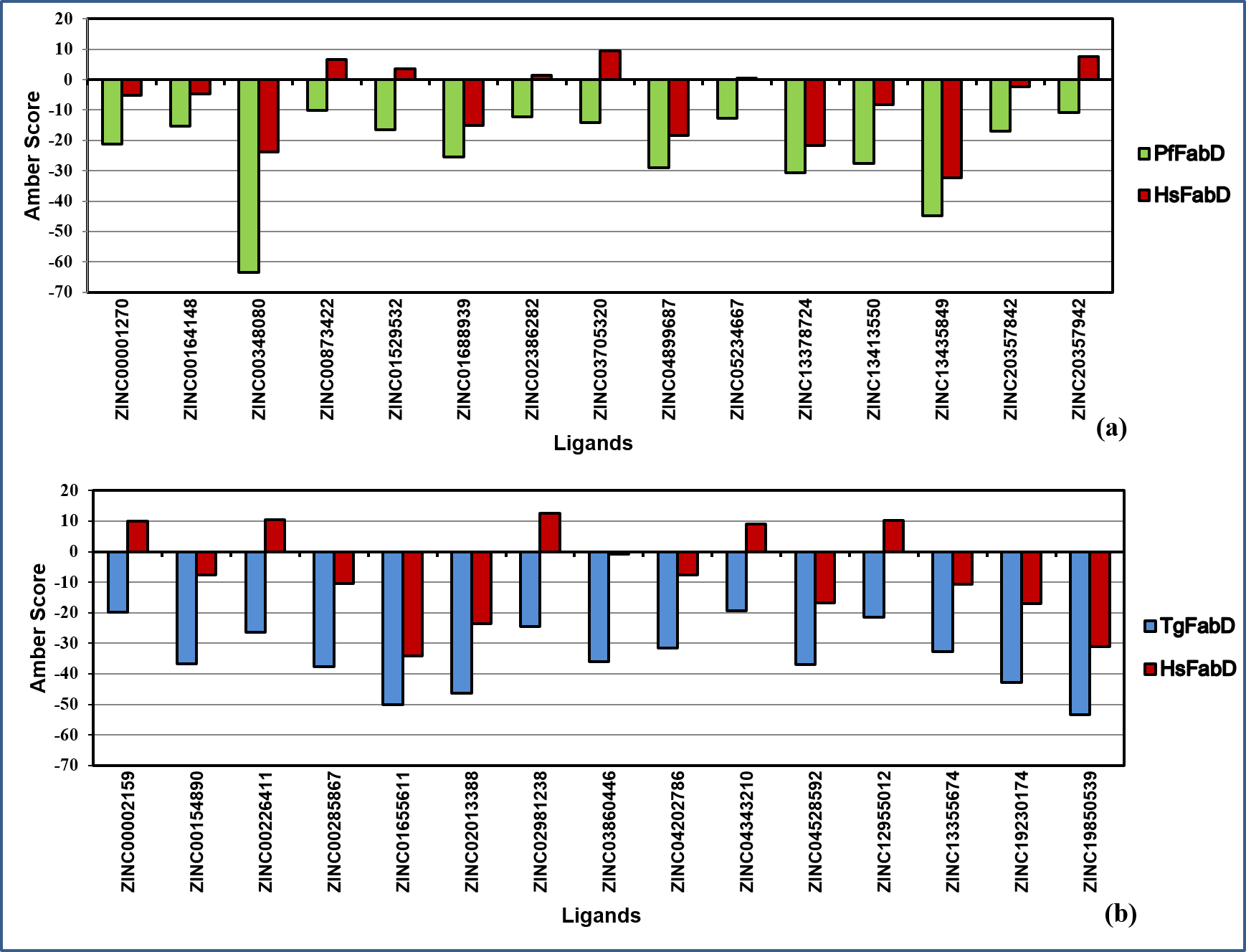

Supplement: S8 Fig — (TIF) [file pone.0141674.s017.tif]

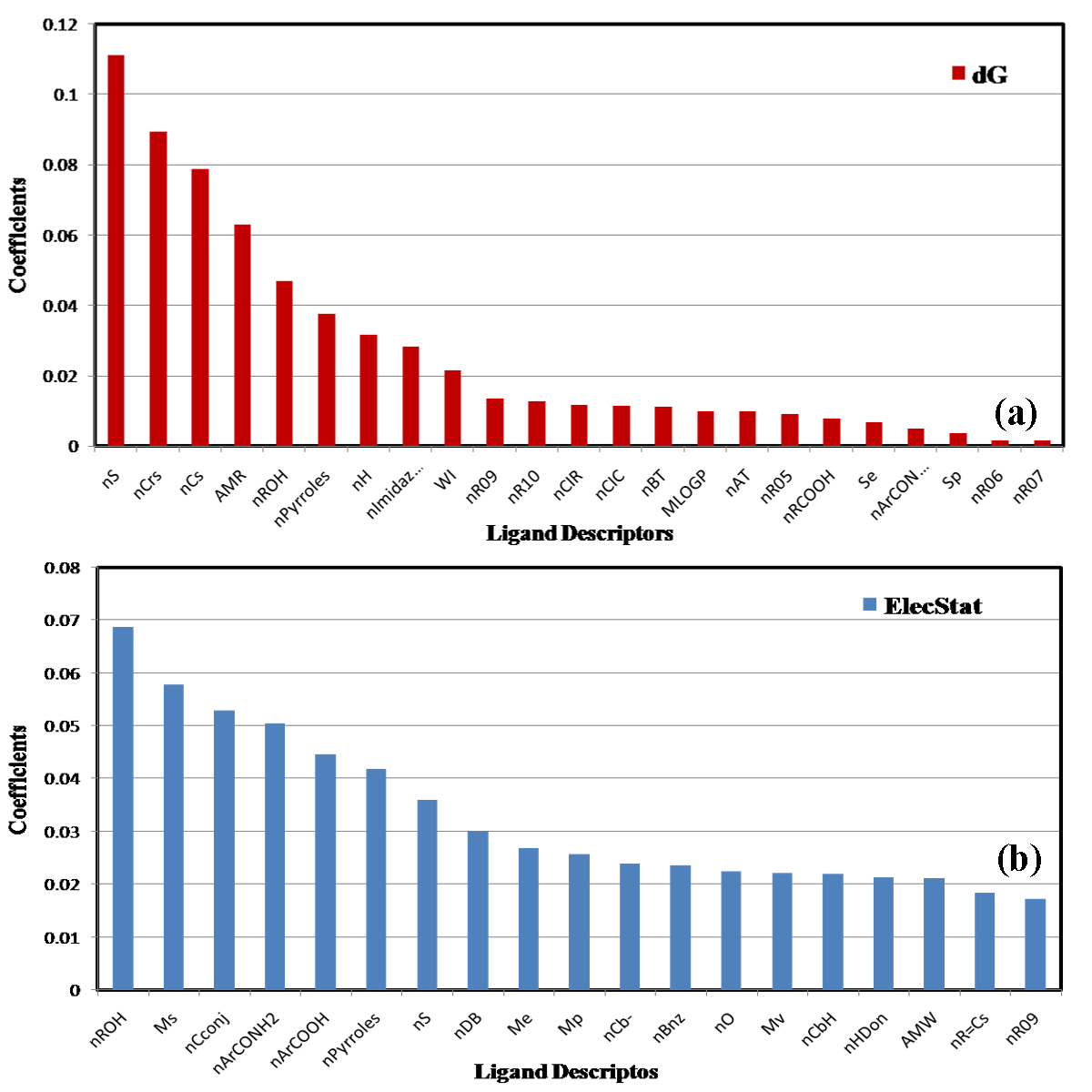

Supplement: S9 Fig — (TIF) [file pone.0141674.s018.tif]

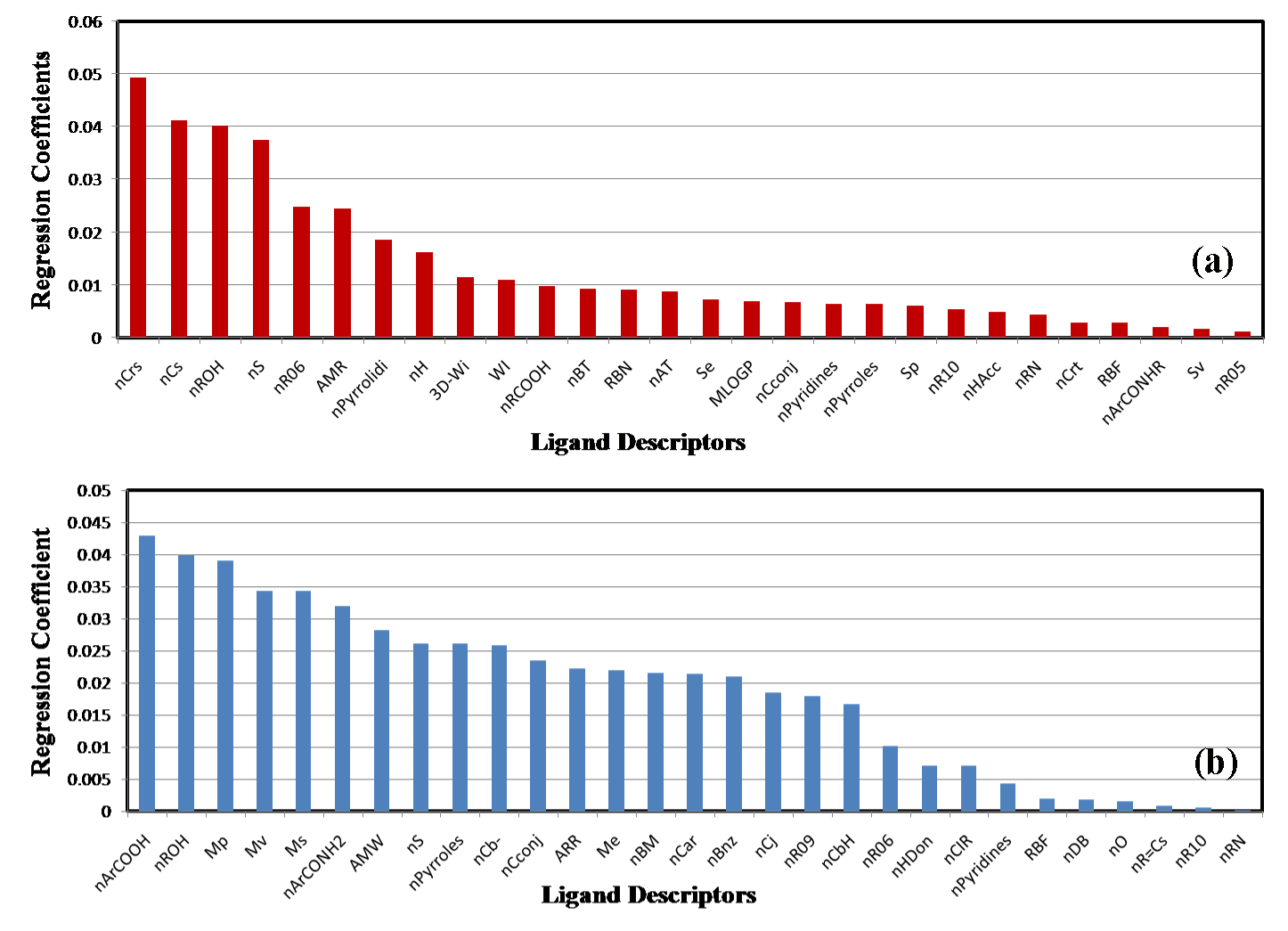

Supplement: S10 Fig — (TIF) [file pone.0141674.s019.tif]

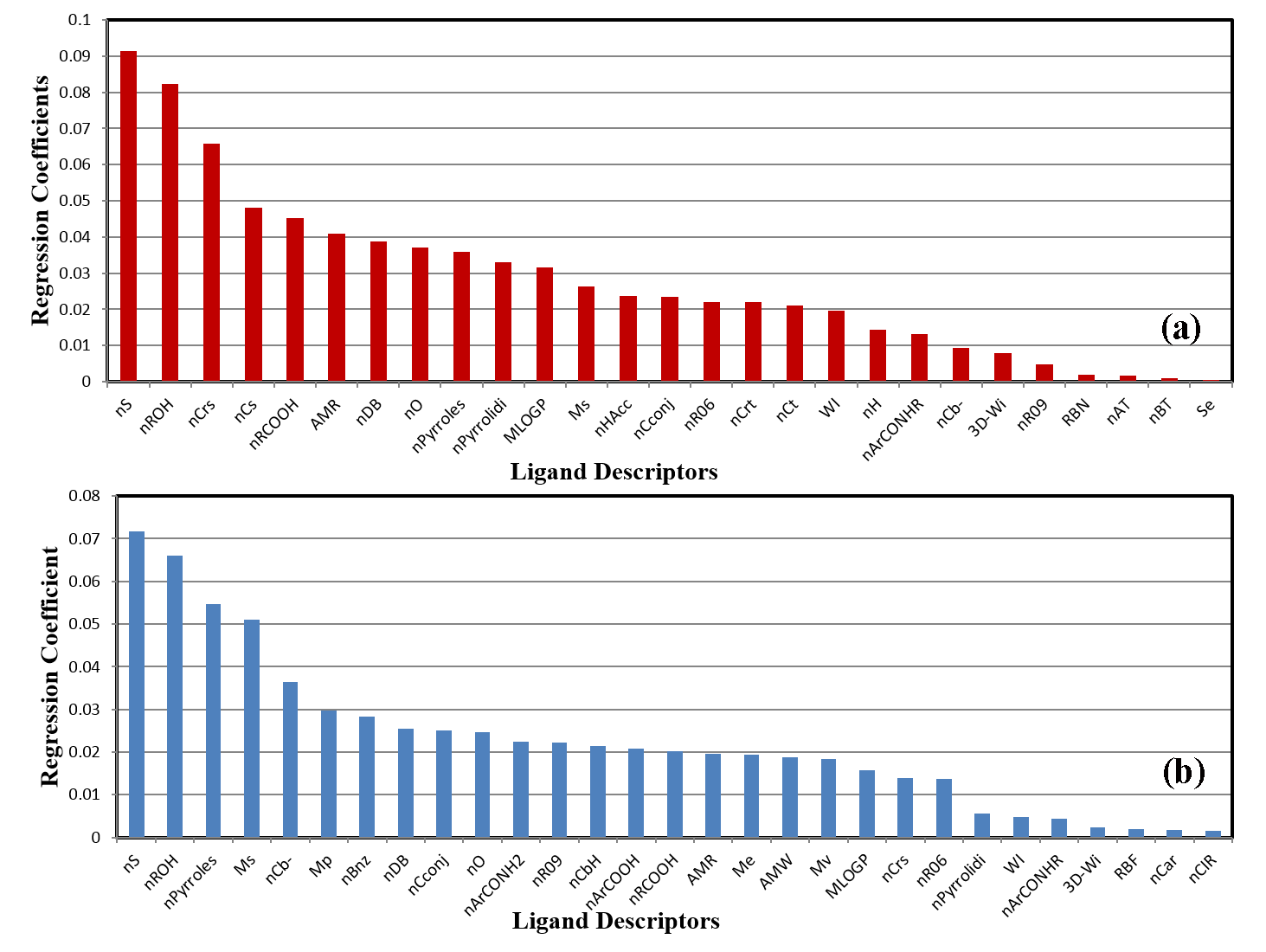

Supplement: S11 Fig — (TIF) [file pone.0141674.s020.tif]

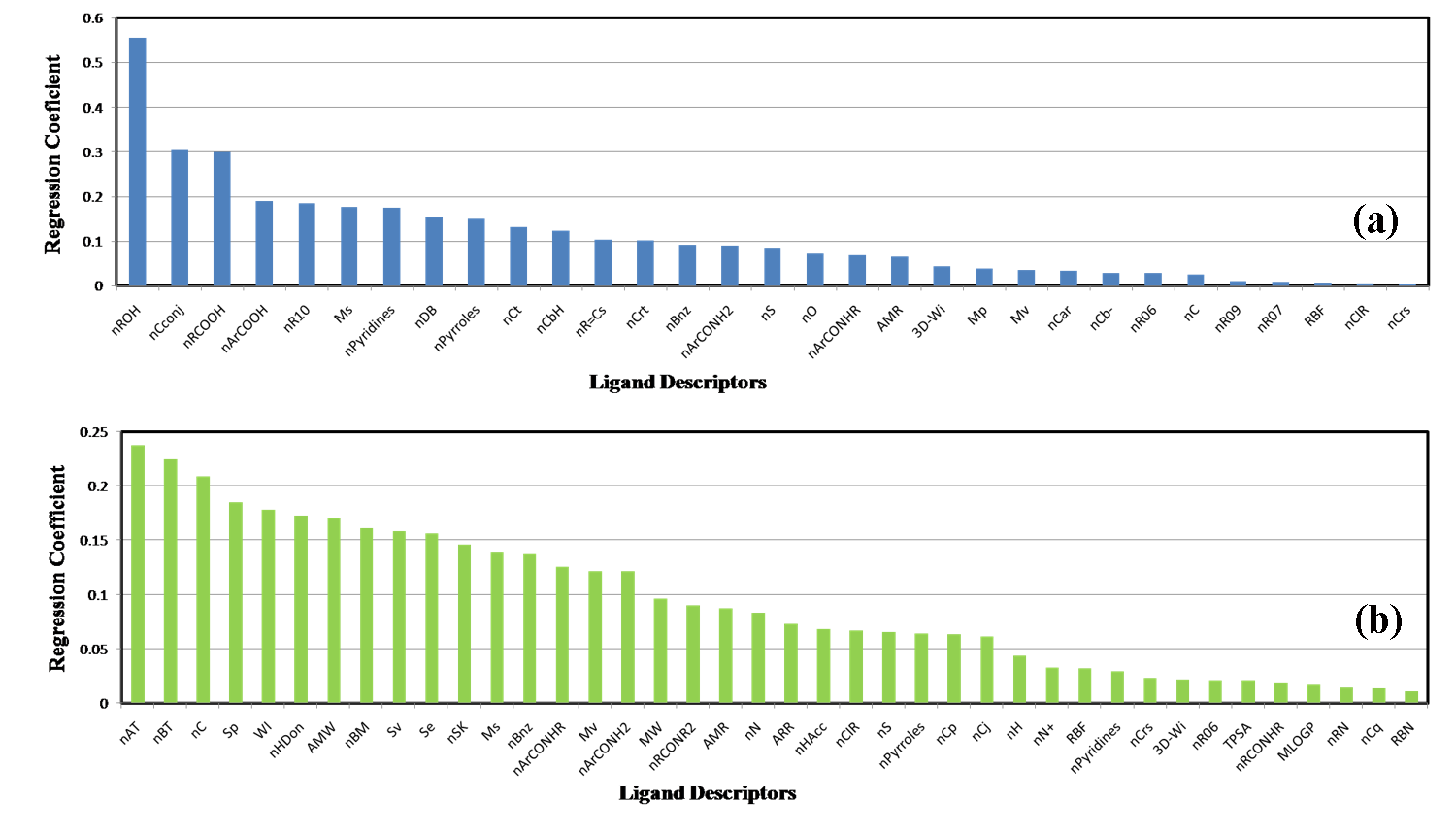

Supplement: S12 Fig — (TIF) [file pone.0141674.s021.tif]

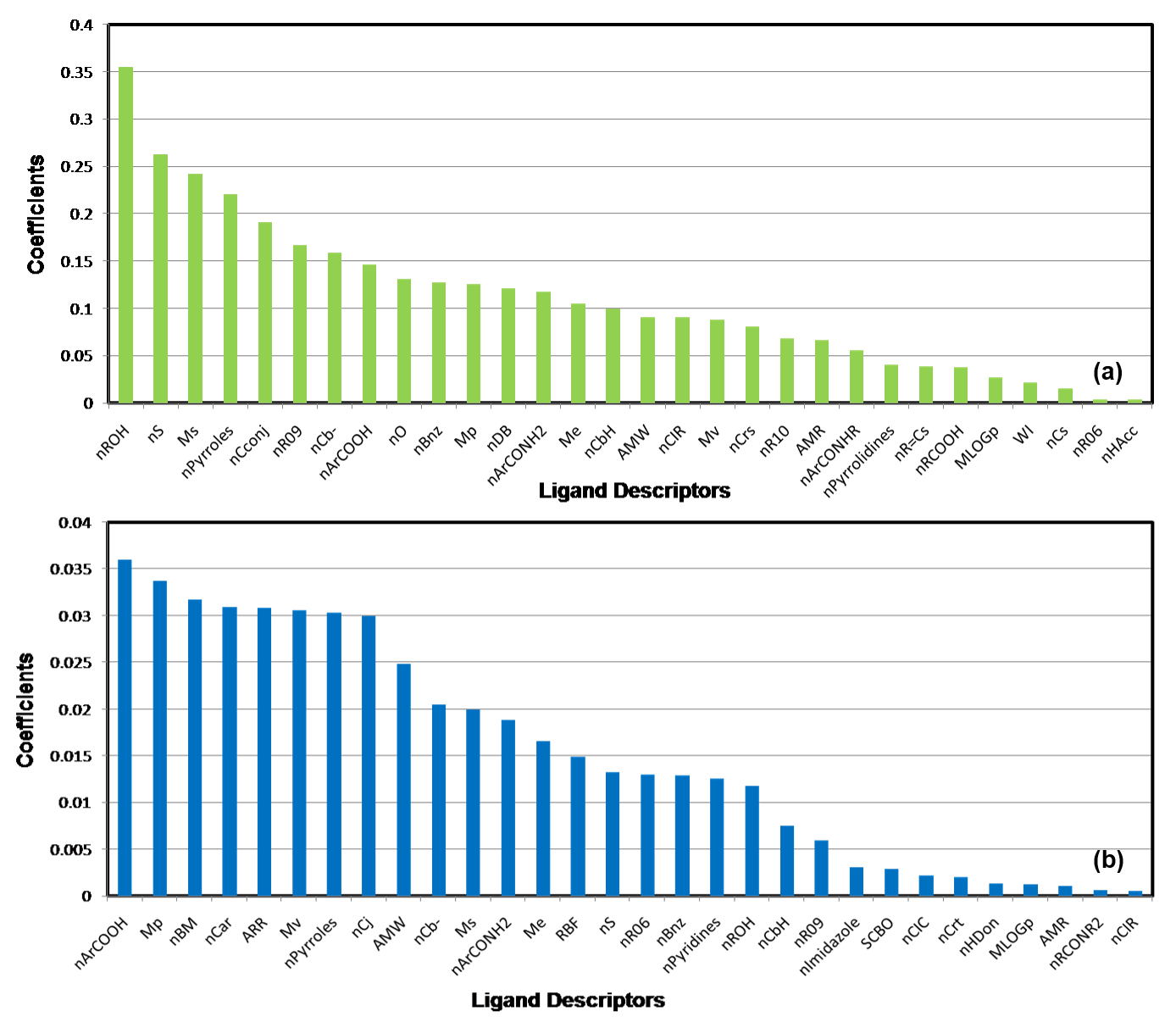

Supplement: S13 Fig — (TIF) [file pone.0141674.s022.tif]

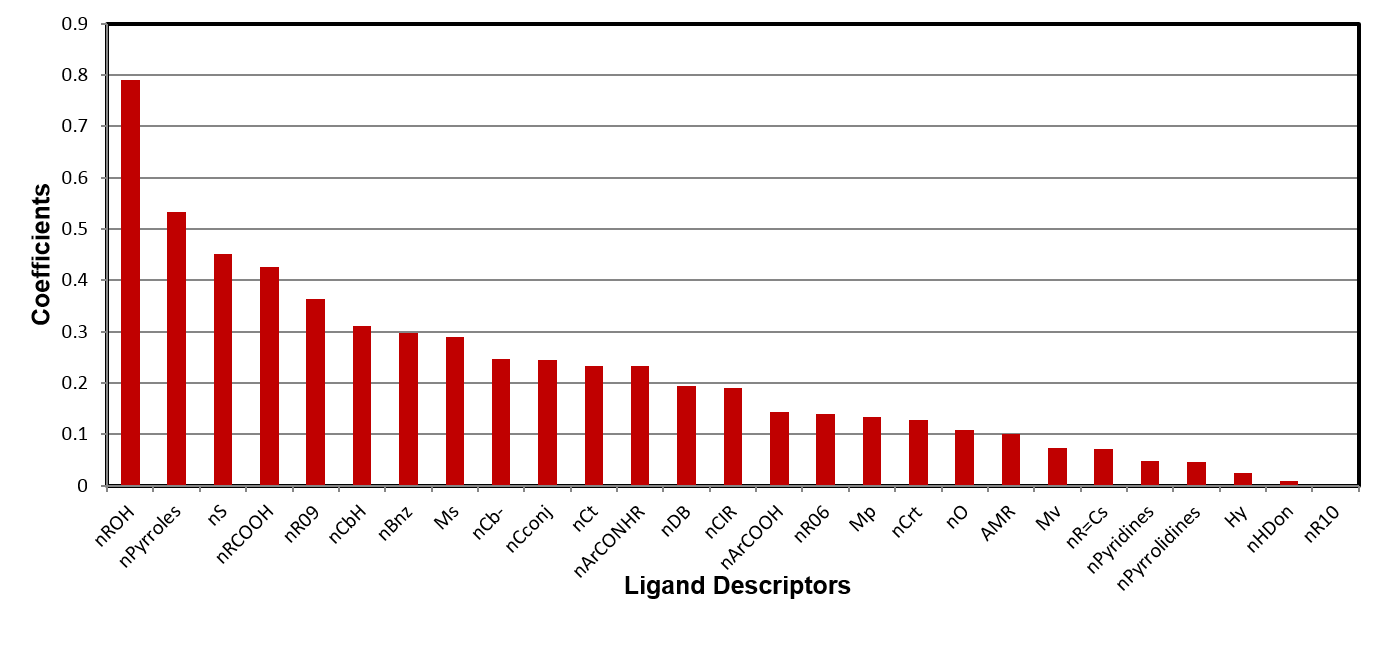

Supplement: S14 Fig — (TIF) [file pone.0141674.s023.tif]
